# Cell-microsphere based living microhybrids for osteogenesis regulating to boosting biomineralization

**DOI:** 10.1093/rb/rbae125

**Published:** 2024-10-29

**Authors:** Zhaofan Hu, Yunyang Zhang, Jingjing Zhang, Ran Zheng, Yang Yang, Fei Kong, Haoran Li, Xinyan Yang, Shuhui Yang, Xiangdong Kong, Ruibo Zhao

**Affiliations:** Institute of Smart Biomedical Materials, School of Materials Science & Engineering, Zhejiang Sci-Tech University, Hangzhou 310000, PR China; Zhejiang-Mauritius Joint Research Center for Biomaterials and Tissue Engineering, Zhejiang Sci-Tech University, Hangzhou 310018, PR China; Institute of Smart Biomedical Materials, School of Materials Science & Engineering, Zhejiang Sci-Tech University, Hangzhou 310000, PR China; Zhejiang-Mauritius Joint Research Center for Biomaterials and Tissue Engineering, Zhejiang Sci-Tech University, Hangzhou 310018, PR China; Institute of Smart Biomedical Materials, School of Materials Science & Engineering, Zhejiang Sci-Tech University, Hangzhou 310000, PR China; Zhejiang-Mauritius Joint Research Center for Biomaterials and Tissue Engineering, Zhejiang Sci-Tech University, Hangzhou 310018, PR China; Institute of Smart Biomedical Materials, School of Materials Science & Engineering, Zhejiang Sci-Tech University, Hangzhou 310000, PR China; Zhejiang-Mauritius Joint Research Center for Biomaterials and Tissue Engineering, Zhejiang Sci-Tech University, Hangzhou 310018, PR China; Institute of Smart Biomedical Materials, School of Materials Science & Engineering, Zhejiang Sci-Tech University, Hangzhou 310000, PR China; Zhejiang-Mauritius Joint Research Center for Biomaterials and Tissue Engineering, Zhejiang Sci-Tech University, Hangzhou 310018, PR China; Institute of Smart Biomedical Materials, School of Materials Science & Engineering, Zhejiang Sci-Tech University, Hangzhou 310000, PR China; Zhejiang-Mauritius Joint Research Center for Biomaterials and Tissue Engineering, Zhejiang Sci-Tech University, Hangzhou 310018, PR China; Institute of Smart Biomedical Materials, School of Materials Science & Engineering, Zhejiang Sci-Tech University, Hangzhou 310000, PR China; Zhejiang-Mauritius Joint Research Center for Biomaterials and Tissue Engineering, Zhejiang Sci-Tech University, Hangzhou 310018, PR China; School of Laboratory Medicine and Bioengineering, Hangzhou Medical College, Hangzhou 311399, PR China; Institute of Smart Biomedical Materials, School of Materials Science & Engineering, Zhejiang Sci-Tech University, Hangzhou 310000, PR China; Zhejiang-Mauritius Joint Research Center for Biomaterials and Tissue Engineering, Zhejiang Sci-Tech University, Hangzhou 310018, PR China; Institute of Smart Biomedical Materials, School of Materials Science & Engineering, Zhejiang Sci-Tech University, Hangzhou 310000, PR China; Zhejiang-Mauritius Joint Research Center for Biomaterials and Tissue Engineering, Zhejiang Sci-Tech University, Hangzhou 310018, PR China; Institute of Smart Biomedical Materials, School of Materials Science & Engineering, Zhejiang Sci-Tech University, Hangzhou 310000, PR China; Zhejiang-Mauritius Joint Research Center for Biomaterials and Tissue Engineering, Zhejiang Sci-Tech University, Hangzhou 310018, PR China; Shengzhou Innovation Research Institute, Zhejiang Sci-Tech University, Shengzhou, Zhejiang 312451, PR China

**Keywords:** living materials, porous microspheres, osteogenesis regulation, biomineralization

## Abstract

Biomineralization-based cell-material living composites *ex vivo* showed great potential for living materials construction and cell regulation. However, cells in scaffolds with unconnected pores usually induce confined nutrient transfer and cell–cell communications, affecting the transformation of osteoblasts into osteocytes and the mineralization process. Herein, the osteoblast-materials living hybrids were constructed with porous PLLA microspheres using a rational design, in which cell-based living materials presented an improved osteoblast differentiation and mineralization model using rationally designed cell-microsphere composites. The results indicated that the microfluidic-based technique provided an efficient and highly controllable approach for producing on-demand PLLA microspheres with tiny pores (<5 μm), medium pores (5–15 μm) and large pores (>15 μm), as well as further drug delivery. Furthermore, the simvastatin (SIM)-loaded porous PLLA microsphere with ε-polylysine (ε-PL) modification was used for osteoblast (MC3T3-E1) implantation, achieving the cell-material living microhybrids, and the results demonstrated the ε-PL surface modification and SIM could improve osteoblast behavior regulation, including cell adhesion, proliferation, as well as the antibacterial effects. Both *in vitro* and *in vivo* results significantly demonstrated further cell proliferation, differentiation and cascade mineralization regulation. Then, the quantitative polymerase chain reaction or histological staining of typical markers, including collagen type I, alkaline phosphatase, runt-related transcription factor 2 and bone morphogenetic protein 2, as well as the calcium mineral deposition staining *in situ*, reconfirmed the transformation of osteoblasts into osteocytes. These achievements revealed a promising boost in osteogenesis toward mineralization at the microtissue level by cell-microsphere integration, suggesting an alternative strategy for materials-based *ex vivo* tissue construction and cell regulation, further demonstrating excellent application prospects in the field of biomineralization-based tissue regeneration.

## Graphical abstract

**Figure:**
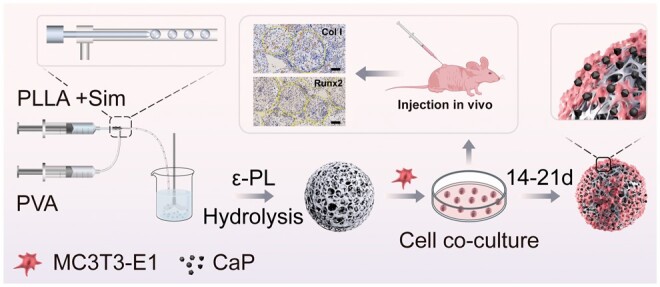

## Introduction

Biomineralization is the primary process for skeleton regeneration [1, 2], which integrates osteoblast infiltration and proliferation within implanted materials, initiating osteoblast differentiation to osteocytes and driving mineralization subsequently. When we consider osteoblast-osteocyte differentiation and mineralization *in vivo*, it generally includes the cell migration into the prosperous area and differentiation to mineralization induced by the microenvironment and active biomolecules [3]. Inspired by this, the implanted scaffolds, such as Poly (lactic acid) (PLA), demonstrated high biocompatibility for osteoblast infiltration and proliferation during bone regeneration [4–8]. However, during mineralization engineering, scaffolds with limited porosity and unconnected pores usually induce confined nutrient transfer and cell–cell communications within implanted materials [9], which inhibits osteoblast viability and information exchange [10], affecting the mineralization process [11, 12].

Currently, the materials-assisted cell hybrid presents a novel insight into regulating cell behavior and function, demonstrating outstanding potential for regenerative medicine [13, 14]. More importantly, cell-material-based living materials provided *ex vivo* techniques to improve the comprehensive understanding by mimicking the way of cell regulation by implanted material *in vivo* [15, 16], which provided a facile strategy to detect cell behavior and biomineralization regulation dynamically. Cell-material complexes need further careful consideration of permeability to achieve living and regulating functions. Recently, the porous microspheres with large specific surface areas have demonstrated great potential for cell proliferation [17, 18], and their highly interconnected pore structure facilitates cell viability and sustained drug release [19–21]. Poly (L-lactic acid) (PLLA), a typical PLA biomaterial, has demonstrated great potential for skeleton repair due to its flexible mechanical properties, biodegradability and biocompatibility [22–24]. When used as microspheres, their confined pore size and unconnected inner structure limited their further osteocyte regulation [25], presenting a critical challenge for osteoblast regulation and mineralization. It was suggested that regulating osteocyte differentiation and mineralization by porous microspheres *in situ* might be preferred [12, 26]. It should be noted that porous microspheres with 100–500 μm in diameter supported fluent nutrient exchange for cell proliferation and communications [21]. Moreover, a pore size of ∼20 μm and its inner connection within the microspheres could improve cell infiltration and accommodation [21]. Inspired by these, it followed a promising strategy for constructing osteocyte-based living materials by porous PLLA microspheres.

**Figure 1.**
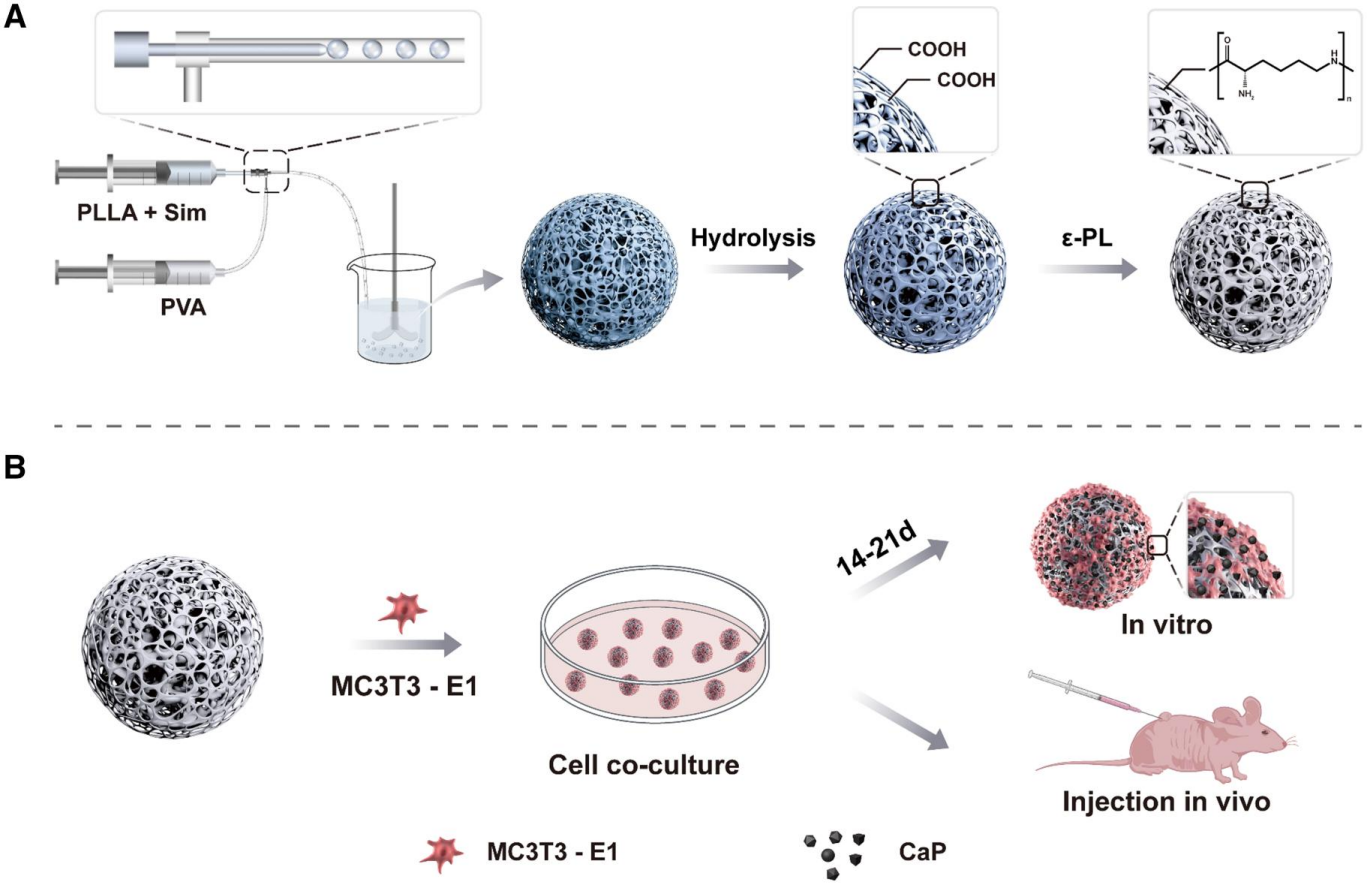
Scheme of the preparation of PPM-SIM-ε-PL and their regulation for osteoblast-implanted living biomaterials.

Herein, the rationally designed porous PLLA microspheres were achieved by microfluidics. To achieve cell regulation, simvastatin (SIM), a typical inducer for osteoblast differentiation [27, 28], was introduced facilely into microspheres using one-step preparation followed by ε-polylysine (ε-PL) surface modification (Figure 1). These achievements presented a novel alternative strategy for porous PLLA microspheres, demonstrating the success of osteoblast hybrids, which provided a comprehensive biomimetic mineralization process involving osteocyte differentiation and mineralization *in situ*, further indicating the success of cell-based living materials construction at microtissue levels.

## Materials and methods

### Materials

PLLA (Mw = 60 kDa) was purchased from (Daigang, China). α-Minimum essential medium (α-MEM) was obtained from Cytiva. Dulbecco’s modified Eagle’s medium was obtained from Gibco. Fetal bovine serum (FBS) was obtained from Ozfan. Calcein and poly(vinyl alcohol) (PVA) were purchased from Aladdin. SIM, ammonium bicarbonate and ε-PL were purchased from Macklin (Shanghai, China). The BCA protein assay kit, alkaline phosphatase (ALP) kit, Alizarin Red Staining kit, BCIP/NBT ALP kit, 1,1′-dioctadecyl-3,3,3′,3′-tetramethylindocarbocyanine perchlorate (DiI), cell viability kit, cell counting kit-8, actin-tracker green-488 and 2-(4-amidinophenyl)-6-indolecarbamidine dihydrochloride were supplied by Beyotime Biotechnology (Shanghai, China). Some elements in graphical abstract, scheme and other figures have permission from the BioRender.

### Preparation of PPM

In this study, we prepared porous PLLA microspheres using microfluidic technology and compared different ammonium bicarbonate concentrations, homogenization rates, W/O solution ratios and PLLA concentrations. The steps were as follows: PLLA powder was dissolved in methylene chloride to form the oil phase, while ammonium bicarbonate and PVA were each dissolved in ultrapure water to form the aqueous phase. Next, the oil and ammonium bicarbonate phases were thoroughly mixed and homogenized for 2 min to ensure the formation of a primary emulsion with homogeneous water and oil phases. Afterward, this primary emulsion was used as the dispersed phase in a microfluidic device with a 1% (w/v) aqueous solution of PVA for both the continuous phase and the collection bath. Porous microspheres were prepared using a coaxial needle equipped with an 18-G outer needle and a 25-G inner needle at a 2-ml/min flow rate for the continuous phase and 0.1 ml/min for the dispersed phase. After droplet formation, the droplets were stirred overnight by mechanical stirring at 220 rpm/min to ensure complete volatilization of dichloromethane. The decomposition of ammonium bicarbonate produced ammonia and carbon dioxide, which formed highly interconnected pore structures inside and on the surface of the microspheres. Finally, the microspheres were washed three times with ultrapure water to remove PVA, and these porous PLLA microspheres (PPM) were preserved by freeze-drying. In this study, PLLA membranes were prepared for water contact angle measurements. The PLLA solution in 2.5% (w/v) DCM was uniformly coated on circular slides (Φ = 40 mm) and the DCM was allowed to evaporate for 12 h at room temperature.

Drug-loaded microspheres (PPM-SIM) were prepared by introducing the SIM into the PLLA solution directly during preparation. For the typical preparation of PPM-SIM, the preparation method was as follows: 0.2 g of PLLA powder, 8 mg∼24 mg of SIM was dissolved in 8 ml of DCM, 3 ml of the above solution was taken, 1 ml of 1% (w/v) aqueous ammonium bicarbonate was added, and homogenization was carried out at a low rate of 5000 rpm for 2 min to obtain the W/O emulsion to be used as the dispersed phase, and the subsequent steps were the same as PPM.

To prepare ε-PL coated microspheres, PPM was immersed in 0.1 M NaOH for 3 min, rinsed and treated with ε-PL solutions of 1, 3 or 5 mg/ml overnight under mechanical stirring (100 rpm). The groups mentioned above, designated PPM-ε-PL(1), PPM-ε-PL(3) and PPM-ε-PL(5), respectively, were then rinsed and freeze-dried. Then, the optimal ε-PL concentration was employed to coat PPM-SIM microspheres, forming the PPM-SIM-ε-PL microspheres.

### Characterizations

The stereoscope (NSZ-608T, China), microscope (Leica, Germany) and scanning electron microscope (SEM) (Zeiss, USA), as well as EDS elemental mapping (Carl Zeiss ultra 55, Germany), were used to characterize the morphology of various samples, and the particle and pore sizes of the microspheres were measured by Nano Measure (*n* = 100). Confocal microscopy (Nikon, Japan) was used to characterize the microstructure inside the microspheres. The chemical composition of samples was detected by X-ray photoelectron spectroscopy (EscaLab 250Xi, Thermo Fisher, USA) and Fourier transform infrared (FTIR) spectroscopy (IS 50, Thermo Electron Scientific Corporation, USA). The crystal structure of the samples was determined using a powder X-ray diffraction (XRD) instrument (Bruker AXS GMBH, USA). The thermal behavior of the samples was analyzed by thermogravimetric analysis (TGA) (HS-101, Heson, China). PLLA films were prepared as a substitution for the water contact angle test (JC2000D2, China). The surface potentials of the samples were analyzed using a solid surface zeta potential analyzer (SurPASS 3, ATU).

### Loading efficiency and encapsulation efficiency

To measure the loading and encapsulation efficiency of SIM, groups of microspheres were dissolved in dichloromethane and diluted to a concentration of 100 μg/ml. The diluted samples were detected using a UV spectrophotometer at 240 nm. The drug loading and encapsulation efficiency were determined:

$$\begin{array}{c} Loading\;Efficiency\;\left(\%\right)=\frac{Weight\;of\;drug\;in\;microspheres}{Weight\;of\;microspheres}*100\\ Encapsulation\;Efficiency\;\left(\%\right)=\frac{Actual\;drug\;loading}{Theoretical\;drug\;loading}*100 \end{array}$$

### Drug release property

Ten milligrams of SIM-loaded microspheres were added to a dialysis bag with a molecular weight cut-off of 10 000 Da and immersed in a centrifuge tube containing 0.5% (v/v) Tween 20 in PBS solution (20 ml, pH = 7.4). Then, they were placed in an oscillating incubator (120 rpm, 37°C). Approximately 3 ml of release medium was removed at specific time points (2 h ∼ 14 days) and replaced with 3 ml of fresh release medium. The SIM characteristic peak was detected at 239 nm using a UV spectrophotometer.

### Biodegradability of PPM

Five milligrams of each group microspheres (PPM, PPM-ε-PL and PPM-SIM-ε-PL) were immersed in 10 ml of PBS and incubated at 37°C with gentle shaking. After 10, 20 and 30 days, the microspheres were washed, dried and weighed (W_t_). The residue percentage was calculated using the formula (*W*_t_/*W*_0_) × 100%, where *W*_0_ represents the initial weight of the microspheres. This process was performed in triplicate for each time point.

### Antimicrobial properties

The antimicrobial properties were detected by co-culturing with *Staphylococcus aureus* (*S. aureus*) and *Escherichia coli* (*E. coli*) with PPM, PPM-ε-PL and PPM-SIM-ε-PL microspheres for 4, 8 and 12 h, respectively. Briefly, 10 mg of PPM, PPM-ε-PL and PPM-SIM-ε-PL microspheres were taken into 24-well plates, and 1 ml *E. coli* (ATCC 25922) and *S. aureus* (ATCC 25923) suspensions were diluted to 1.0 × 10^4^ CFU/ml and added into each well. The untreated bacteria were used as a blank control, and the bacterial co-culture suspensions were incubated at 37°C for 4, 8 and 12 h. The OD bacterial suspension was separated and measured at 600 nm, and the viability of the bacteria in each group was calculated by the formula: *D* sample/*D* control×100%. *D* sample and *D* control denote the absorbance values of the samples and the control.

### Cytotoxicity test

The biocompatibility of PPM was investigated using the CCK8 method. To assess the cytotoxicity of each group of microspheres, the microspheres were sterilized for 2 h by UV disinfection. Then, 20 mg microspheres were immersed in 5 ml of culture medium (10% FBS and 1% penicillin-streptomycin) and placed into an incubator (37°C, 5% CO_2_) for 24 h. Subsequently, the microsphere supernatant was filtered through a 0.22-μm filter in two rounds. The microsphere extract was diluted to five concentration gradients of 2.5, 1, 0.5, 0.25 and 0.125 mg/ml. L929 and MC3T3-E1 cells were seeded into a 96-well at a density of 2.0 × 10^4^ cells/ml and incubated for 24 h. After 24 h, the medium was replaced with 100 μl of microsphere extract for each group and continued for 24 h incubation. Ten microliters of CCK8 were added to each well, and after 1 h of incubation, the absorbance at 450 nm was measured in a microplate reader.

### Cell viability assay

The PPM was soaked in 75% alcohol for 30 min, and the microspheres were washed with PBS 3 times, then MC3T3-E1 cells (1 × 10^6^ cell/well) and microspheres (20/well) were added into 5 ml centrifuge tubes treated with an anti-adhesion solution. The tubes were blown sufficiently using a pipette to make the cells and microspheres in full contact. Then, the centrifuge tubes were incubated in an incubator for 2 h (37°C, 5% CO_2_), and 70 μm filters were used to remove the unattached cells. The cell-microsphere complexes were transferred to 24-well plates for further incubation, and the medium was changed every two days. The cell microsphere complexes were stained by Live/Dead Kit after 1, 3 and 5 days of incubation and were observed using a confocal laser scanning microscope (CLSM). Furthermore, MC3T3-E1 cell-laden microspheres were suspended in a 2-ml culture medium and transferred into a 24-well plate for continued culture. Cell migration and proliferation from microspheres to the plate were observed using an optical microscope at indicated intervals. ImageJ software was used to quantify the cell area percentage at each time point.

### Osteogenesis and mineralization regulation

MC3T3-E1 cells were implanted into the microspheres and cultured in α-MEM for 5 days before replacing the original medium with an osteogenic medium consisting of α-MEM supplemented with 10% FBS, 1% double antibody, 50 μg/ml ascorbic acid, 10 mM sodium β-glycerophosphate and 10 nM dexamethasone.

#### ALP activity

According to the instruction manual of the ALP assay kit, after lysis and centrifugation of the cell microsphere complex, 50 μl of sample and 50 μl of chromogenic substrate were added into 96-well plates and mixed thoroughly. The reaction was terminated by adding 100 μl termination solution to each well after incubation for 30 min at 37°C. Absorbance was measured at 405 nm. A BCA protein concentration assay kit was used to quantify the protein concentration in the supernatant.

#### BCIP/NBT ALP staining

Three sets of cell microsphere complexes after 14 days of co-culture were transferred into 24-well plates. After discarding the culture medium, the plates were washed twice with PBS solution. Next, 4% paraformaldehyde was added to the 24-well plate and fixed for 10 min. After discarding the fixation solution, it was washed three times with ultrapure water. Then, the plates were stained at room temperature for 12 h using the BCIP/NBT ALP chromogenic kit and photographed.

#### Real-time polymerase chain reaction analysis

Following 14 days of culture in an osteogenic induction medium, the three groups of cell-material complexes were collected, and total RNA was extracted using a Trizol reagent (Thermo Fisher, USA). Expression of four osteogenesis-related genes (collagen type I [Col I], ALP, Runt-related transcription factor 2 [Runx2] and bone morphogenetic protein 2 [BMP2]) was analyzed by quantitative reverse transcription polymerase chain reaction (PCR) using SYBR Green Master Mix (Applied Biosystems) on a CFX384 Real-Time PCR Detection System (Bio-Rad, USA) [29]. Gene expression was analyzed using the ΔΔCT method by being normalized with GAPDH. The primers used in this study are listed in Table S1.

#### Staining and analysis

Three sets of cell microsphere complexes were transferred into 24-well plates after 14 and 21 days of co-culture. After discarding the culture medium, they were washed twice with PBS solution. Next, 4% paraformaldehyde was added to the 24-well plates and fixed for 10 min. After discarding the fixation solution, it was washed three times with ultrapure water. Then, Alizarin Red Staining solution was added to the 24-well plates, stained at 37°C for 30 min, and washed three times with PBS. Subsequently, 10% cetylpyridinium chloride was added to 24-well plates and incubated at 25°C for 2 h. Finally, 90 μl of supernatant from each well was transferred to 96-well plates individually, and absorbance was measured at 562 nm. As for calcium mineralization mineral staining, the cell microsphere complexes, co-cultured for 14 days, were incubated with 10 μM calcium dye for 5 min and imaged using confocal microscopy. For Von Kossa staining, samples were fixed after being co-cultured for 21 days, incubated in 5% silver nitrate solution under UV light for 30 min, rinsed and treated with 5% sodium thiosulfate for 5 min. Calcium deposits appeared black or dark brown under microscope examination [30].

### *In vivo* subcutaneous ectopic ossification

MC3T3-E1 cells were seeded onto the three groups of microspheres (PPM, PPM-ε-PL and PPM-SIM-ε-PL) at a density of 1 × 10^6^ cells per 10 mg of microspheres. The cell-laden microspheres were cultured in a growth medium for 24 h to allow cell attachment. Subsequently, 10 mg of each type of cell-laden microsphere was mixed with 0.2 ml of Matrigel (Corning, USA) and subcutaneously injected into the dorsal region of 6-week-old nude mice [31]. The Matrigel alone (Matrigel group) and Matrigel/cell integrated treatments (Matrigel+cell group) served as controls. Animal experiments were conducted under the approval of the ethical review committees of Zhejiang Sci-Tech University (approval number: 20240725-01).

The mice were euthanized 4-week post-implantation, and the implants were harvested and fixed in 4% paraformaldehyde for 24 h, dehydrated through a graded ethanol series and embedded in paraffin. Serial sections (∼4 μm thick) were prepared for histological and immunohistochemical analyses. Hematoxylin and eosin (H&E) staining was performed to evaluate general tissue morphology and cell distribution. Masson’s trichrome staining was used to assess collagen deposition and tissue organization. Alizarin Red S staining was conducted to visualize calcium deposition and mineralization. For immunohistochemical analysis, sections were deparaffinized, rehydrated and subjected to antigen retrieval using citrate buffer (pH 6.0). Endogenous peroxidase activity was quenched with 3% hydrogen peroxide. After blocking with 3% bovine serum albumin, the sections were incubated with primary antibodies against Col I (1:400, Cell Signaling) and Runx2 (1:600, Affinity) overnight at 4°C. The sections were then incubated with HRP-conjugated secondary antibodies and developed using a 3,3′-diaminobenzidine substrate. Counterstaining was performed with hematoxylin. All stained sections were scanned and imaged using a scanner (KFBio KF-PRO-120, China).

### Statistical analysis

The results are expressed as mean±SD and the significance of differences was analyzed by *t*-test. Differences were statistically significant at *P < 0.05, **P < 0.01, ***P < 0.001 and ****P < 0.0001. Statistical analyses and plots were performed using OriginPro or GraphPad Prism.

## Results and discussion

### Synthesis and characterization of PPM

Particularly, open porous microspheres with the size of the microspheres at a range of 400 ∼ 500 µm with pore size at ∼20 µm, maintaining the exchange of oxygen, nutrients and metabolites, have a significant impact on cell proliferation and differentiation [32, 33]. To improve the inner connection, the ammonium bicarbonate was used as pore former typically [34], and it should be noted that variations in parameters, including ammonium bicarbonate concentration, homogenization rate, PLLA concentration, water-to-oil (W/O) ratio, could influence microspheres morphology [35–37], which were further investigated (Table S2).

**Figure 2.**
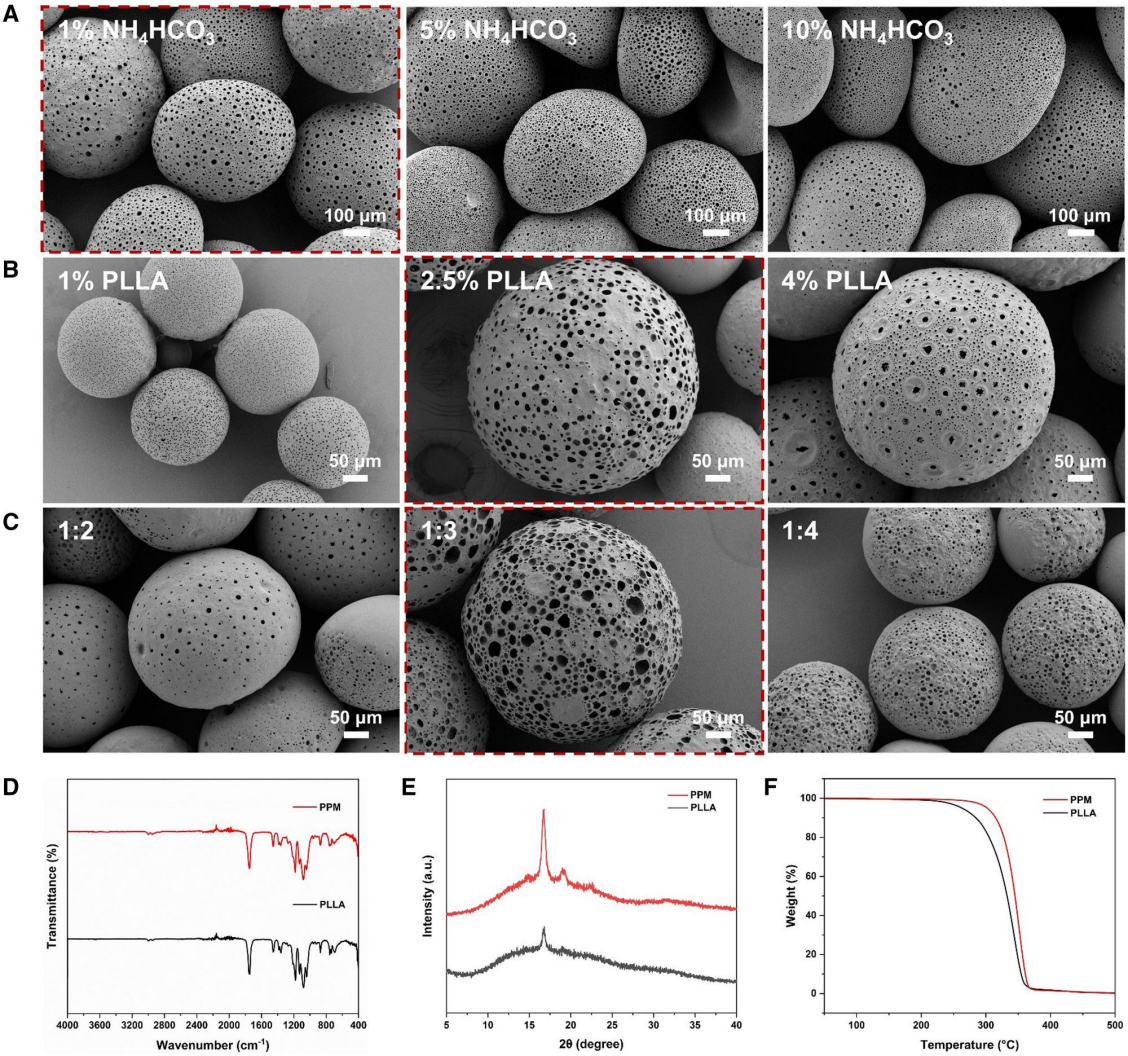
Synthesis and characterization of PPM. (**A**) SEM images depicting PPM synthesized at different NH_4_HCO_3_ concentrations. (**B**) SEM images showing PPM with varying concentrations of PLLA. (**C**) SEM images of PPM produced with different water-to-oil (W/O) ratios. (**D**) IR spectral mapping for characterization of PLLA and PPM. (**E**) XRD spectral mapping to characterize PLLA and PPM. (**F**) TGA for detailed characterization of PLLA and PPM.

To achieve the interconnectivity within PPM, ammonium bicarbonate (NH_4_HCO_3_) was introduced into the preparation system, and the results demonstrated that 1% NH_4_HCO_3_ could induce the uniform PPM with the average porous pore size at ∼12 µm compared with 5% NH_4_HCO_3_ and 10% NH_4_HCO_3_ with the average porous pore size at ∼9 and 8 µm, respectively (Figure S2A). Higher NH_4_HCO_3_ concentrations were associated with significant PPM inhomogeneity, as confirmed by SEM analysis (Figure 2A). We hypothesize that this inhomogeneity is due to reduced droplet viscosity at higher NH_4_HCO_3_ concentrations, which facilitates droplet coalescence during dichloromethane volatilization. Furthermore, an increased homogenization rate led to a decreased pore size of microspheres (Figure S1), this inverse relationship can be attributed to the enhanced shear forces at higher homogenization speeds, which effectively disperse the aqueous ammonium bicarbonate solution into progressively smaller droplets. These diminutive emulsion droplets subsequently give rise to constrained internal spatial structures within the microspheres. Notably, a homogenization rate of 5000 rpm for 2 min yielded microspheres with an average pore size of ∼19 µm (Figure S2B). In addition, PLLA microsphere size decreased to ∼205 µm at a PLLA concentration of 1% (Figure 2B), and the particle size of microspheres was similar both in 2.5% PLLA and 4% PLLA, but with a pore size at ∼15 and ∼9 µm, respectively (Figure S2C). The reduction in pore size may be attributed to the formation of a rapidly curing outer layer on the surface of the microspheres by the high concentration of PLLA. This outer layer restricts the diffusion of gases, allowing them to escape only from specific weak points. As for the water-to-oil (W/O) ratio, compared with the 1:2 and 1:4 groups with a decrease in pore size, the 1:3 group showed reproducible pore size (Figure 2C and Figure S2D). Such results revealed synthesis conditions in detail for producing the on-demand microspheres with tiny pores (<5 μm), medium pores (5–15 μm) and large pores (>15 μm) (Figure S3). Furthermore, the optimized conditions, a 1% NH_4_HCO_3_ concentration, a homogenization rate of 5000 rpm and a 2.5% PLLA with a 1:3 water-oil ratio, were used to prepare microspheres (pore size >15 µm) for subsequent studies including drug loading, *in vitro* and *in vivo* biomineralization regulation. Besides, FTIR analysis confirmed that the microfluidic process showed an ignorable influence on the PLLA material’s chemical properties (Figure 2D). Notably, XRD analysis (Figure 2E) showed an improved crystal phase due to the volatilization-based crystallization, subsequently inducing improved thermal stability (Figure 2F), which was in accordance with the previous results [38].

**Figure 3.**
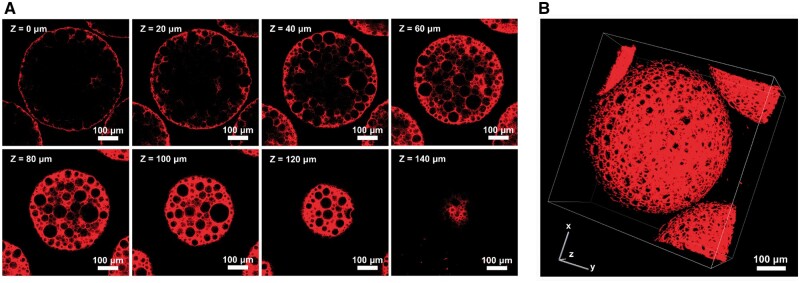
Internal structure of PPM by confocal microscopy. (**A**) Z-stack images (0–140 μm depth) showing DiI dye distribution (red). (**B**) 3D reconstruction revealing interconnected porous structure.

To access the microstructure within PPM, the DiI was used to label PLLA for further CLSM characterization (Figure 3A), and the results exhibited an apparent porous structure within PPM with the average pore size at ∼36 μm, which would be suitable for the following cell infiltration and proliferation [39]. The 3D z-stack results further confirmed the highly interconnected microstructure within microspheres (Figure 3B), which further suggested the advantages of ammonium bicarbonate-based decomposition strategy for interconnected pore construction [35]. These results indicated comprehensive conditions for porous PLLA microsphere preparations and the influences of the material’s properties. It should be noted that no ingredient or chemical modification was introduced into the PLLA, further presenting a successful and facile strategy for reaching PPM.

### PPM-based functional modification

**Figure 4.**
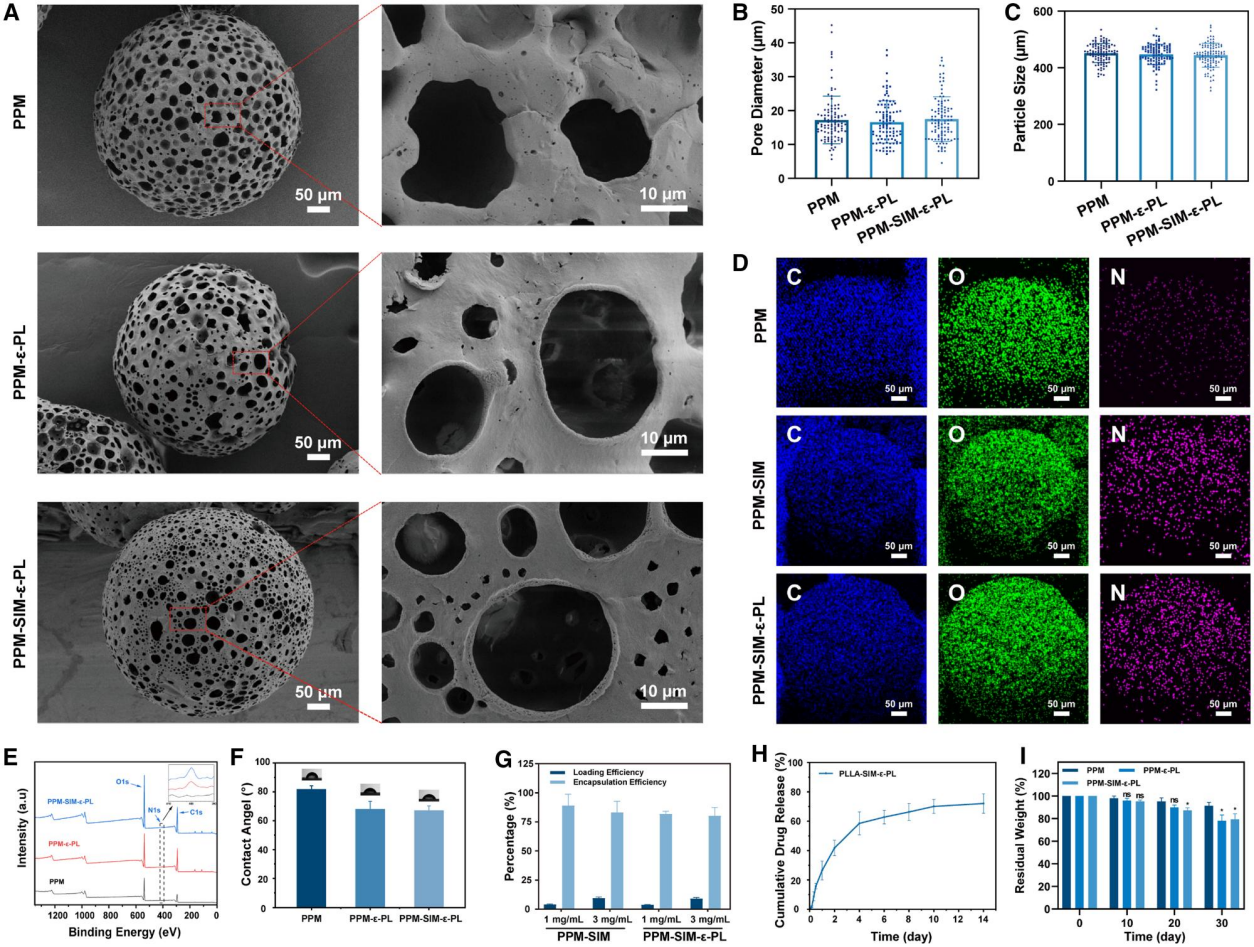
Characterization of drug-loaded coated composite microspheres. (**A**) SEM imaging of PPM, PPM-ε-PL and PPM-SIM-ε-PL. (**B**) Pore diameter distribution of the three groups of microspheres. Pore size measurements are based on SEM images (*n* = 100). (**C**) Particle size distribution of the three groups of microspheres. Particle size measurements are based on microscope images (*n* = 100). (**D**) Elemental mapping images of PPM, PPM-ε-PL and PPM-SIM-ε-PL. (**E**) X-ray photoelectron spectroscopy (XPS) was used to analyze the chemical elements of PPM, PPM-ε-PL and PPM-SIM-ε-PL. (**F**) Water contact angle detection of different substrates (*n* = 3). (**G**) Results of loading efficiency and encapsulation efficiency measurements of PPM-SIM and PPM-SIM-ε-PL. (**H**) Release curve of SIM from PPM-SIM-ε-PL (*n* = 3). (**I**) Residual mass of microspheres during degradation.

To achieve cell microsphere composites for cell regulation, the PPM microspheres loaded with SIM or alone were modified by ε-PL using a NaOH-based hydrolysis approach [40, 41]. The influence of ε-PL concentrations (1, 3 and 5 mg/ml) on microsphere surface properties was investigated (Figure S4). To achieve an optimal balance between efficacy and cost-effectiveness, 3 mg/ml ε-PL (PPM-ε-PL(3)) was selected as the optimal formulation for subsequent experiments. Morphological characterization revealed that the ε-PL coating had almost negligible influence on the microspheres (PPM-ε-PL) compared with the PPM (Figure 4A). Furthermore, the SIM-loaded microspheres (PPM-SIM-ε-PL) depicted a similar morphology with PPM-ε-PL microspheres and PPM, and the average particle size and pore size at 454 and 17.27 μm (PPM), 444 and 16.66 μm (PPM-ε-PL), 441 and 17.51 μm (PPM-SIM-ε-PL), respectively (Figure 4A–C), indicating the SIM loading and ε-PL surface modification showed almost no influences to the preparation of microspheres. The IR spectra of PPM-ε-PL and PPM-SIM-ε-PL show an additional characteristic peak at 1560 cm^−1^, which belongs to the amide II (-NH-) band of ε-PL (Figure S5), demonstrating a successful modification of ε-PL onto PPM and PPM-SIM. A solid surface potential of PPM-ε-PL treated by different concentrations of ε-PL (1, 3 and 5 mg/ml) revealed that the average surface potentials were −40.1 mV for PPM, −31.0 mV for PPM-ε-PL(1), −26.5 mV for PPM-ε-PL(3) and −24.6 mV for PPM-ε-PL(5) (Figure S4), further confirming the successful adhesion of ε-PL to microsphere surfaces (PPM-ε-PL). To further verify the successful coating of ε-PL, elemental mapping and X-ray photoelectron spectroscopy (XPS) analysis were conducted on the microspheres (Figure 4D and E). Elemental mapping revealed a uniform distribution of nitrogen (N) elements within PPM-ε-PL and PPM-SIM-ε-PL samples, which was absent in unmodified PPM. At the same time, XPS analysis further corroborated these findings by exhibiting distinct N1s peaks in the spectra of PPM-ε-PL and PPM-SIM-ε-PL but not in PPM, reconfirming the success of ε-PL modification. In addition, the contact angle shifts from ∼80° of PPM to ∼65° of PPM-SIM-ε-PL (Figure 4F), indicating that the ε-PL modification could improve the material hydrophilia, which was essential for cell seeding and adhesion [41]. Moreover, the microfluidic process presented a typical approach for SIM loading both at low (1 mg/ml) and high concentration (3 mg/ml), and the results revealed that even under a high concentration, it still maintained a high encapsulation efficiency at ∼83.02% with the loading efficiency at ∼8.89% (Figure 4G, PPM-SIM, 3 mg/ml). When loaded with a high concentration of SIM (3 mg/ml), there is a significant structure collapse of the microspheres (Figure S6). Therefore, the 1-mg/ml loaded microspheres would be used for following drug release tests and living materials construction. Besides, the ε-PL modification process almost showed ignorable influences for the loading and encapsulation efficiency (Figure 4G, PPM-SIM-ε-PL), and it exhibited a controlled drug-released pattern (Figure 4H). In addition, a 30-day degradation study was performed to evaluate the long-term stability of the microspheres (Figure 4I). All three microspheres showed gradual degradation over time, with PPM-ε-PL and PPM-SIM-ε-PL showing significantly higher degradation rates than PPM at day 30 (residual weights ∼80% vs ∼90%). The enhanced degradation of ε-PL coated microspheres can be attributed to the increased hydrophilicity, inducing a high water penetration and accelerating PLLA hydrolysis. Notably, the incorporation of SIM did not significantly alter the degradation profile. These results indicate the controlled degradation of the microspheres, suggesting their potential suitability for long-term sustained-release applications.

The antimicrobial properties of microspheres were evaluated against *S. aureus* and *E. coli* over 12 h (Figure S7). It demonstrated that the PPM-SIM-ε-PL showed significant antibacterial activity against *S. aureus* with an inhibition rate of ∼40% at 12 h. In contrast, PPM-ε-PL exhibited slight antibacterial effects with inhibition to ∼10% at 12 h, and the PPM alone had insignificant antibacterial efficacy. These results indicate that the incorporation of SIM conferred effective activity against *S. aureus*, suggesting that the microsphere system demonstrated great potential for antibacterial.

### Construction of cell-material composites

**Figure 5.**
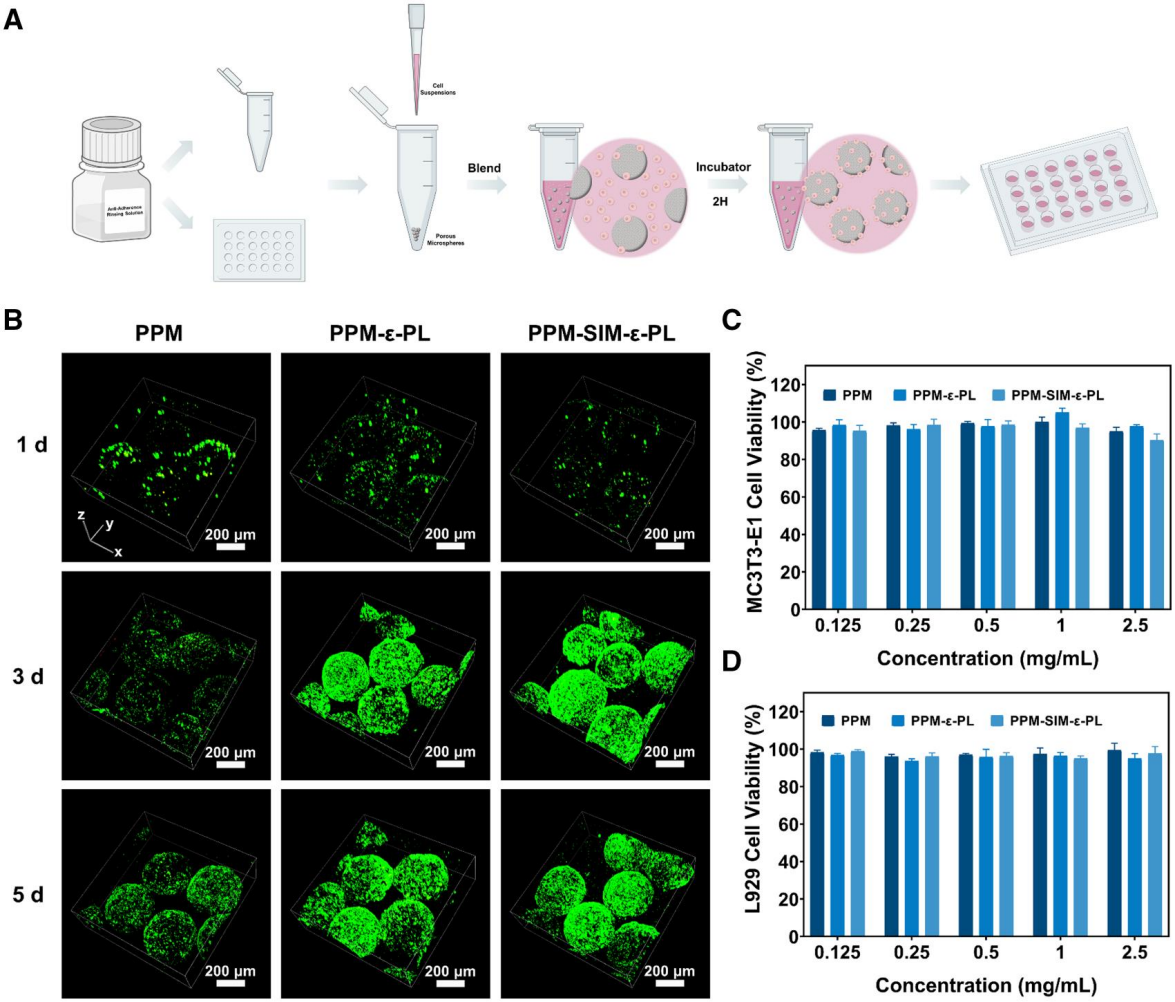
Construction of cell-material composites and evaluation of their biocompatibility. (**A**) Scheme of cell-material composites preparation. (**B**) Live/dead staining of MC3T3-E1 cells cultured within microspheres for 1, 3 and 5 days. (**C**) Cell viability of MC3T3-E1 under microsphere extracts at 24 h. (**D**) Cell viability of L929 under microsphere extracts at 24 h.

Given the success of functional modification of PPM, osteoblast-osteocyte shift-based biomineralization was used as the typical model for PPM-based living materials construction and cell regulation. Briefly, to achieve the cell-microsphere living composites, the 5-ml centrifuge tubes and 24-well plates were treated with an anti-adhesive solution, and subsequently, microspheres were added into the tube followed by adding MC3T3-E1 suspension (5 × 10^6^ cells/ml), and the mix was continued for 2 h incubation (37°C, 5% CO_2_). Then, the unattached cells were removed using a 70-μm filter, and the cell-material composites were transferred to 24-well plates for further cultivation (Figure 5A). Viewed from the SEM, the cells in the PPM-ε-PL and PPM-SIM-ε-PL groups exhibited normal spreading compared with the cells in the PPM group (Figure S8), indicating the advantage of ε-PL modification for cell adhesion [42]. Moreover, the viability of cells within microsphere groups was evaluated using live/dead staining at 1, 3 and 5 days. The results indicated that the PPM-ε-PL and PPM-SIM-ε-PL groups showed significant cell proliferation compared with the PPM alone group (Figure 5B; Figures S9 and S10). Besides, the cell viability at ∼95% under microsphere extracts further confirmed the biocompatibility of all microspheres (Figure 5C and D). These results indicated a brief method of implanting cells into microspheres, inducing cell adhesion and proliferation, and then achieving osteoblast-based living materials.

**Figure 6.**
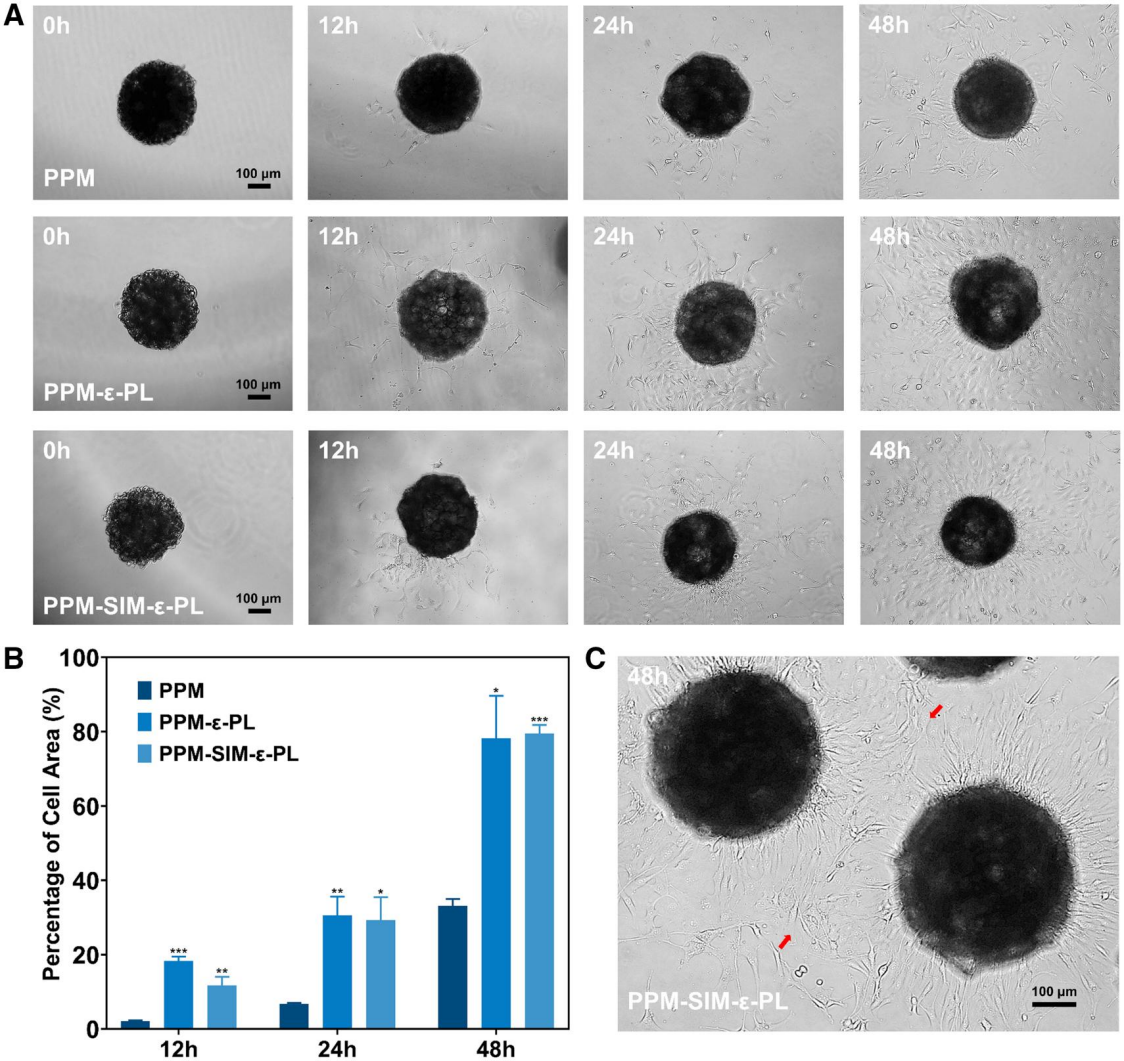
Cell proliferation and spreading assay. (**A**) After injecting microspheres loaded with cells into 24-well plates, MC3T3-E1 cell proliferation and spreading were monitored over time. (**B**) Quantitative analysis of cell area relative to PPM (*n* = 3). (**C**) Proliferation of cells in the PPM-SIM-ε-PL group after 48 h.

Building the connection with the surrounding microenvironment was the typical feature of the living materials [14]. To gain further insight into the proliferative behavior of MC3T3-E1 cells, cell-microsphere complexes were cultured in 24-well plates, with cell migration and proliferation monitored via microscopy. Microscopy and confocal imaging confirmed similar initial cell attachment across all microsphere groups (Figure S11). Through timed monitoring, osteoblasts spreading from the microspheres to the surrounding area were observed, involving cell proliferation and migration [43–45]. It should be noted that cell-microsphere complexes were firmly fixed on the seeding location by the cell migration and proliferation-induced connection between the microspheres and the petri dish (Figure 6A). Quantitative analysis revealed that the area occupied by cells on the dish in the ε-PL modified microsphere group reached 29.96% at 24 h and 78.87% at 48 h, compared with only 6.69% and 33.1% in the PPM group at the same time points, respectively (Figure 6B). This rapid cell spreading provided a living and quick connection between the microspheres and surrounding microenvironments (Figure 6C). These results suggest that the ε-PL modification enhances cell proliferation and the ability of cells to spread from the microspheres, facilitating connections with the surrounding environment. This behavior indicates that these cell-microsphere complexes can be considered dynamic living materials capable of rapid environmental interactions.

### Osteoblast-osteocyte regulation and mineralization

**Figure 7.**
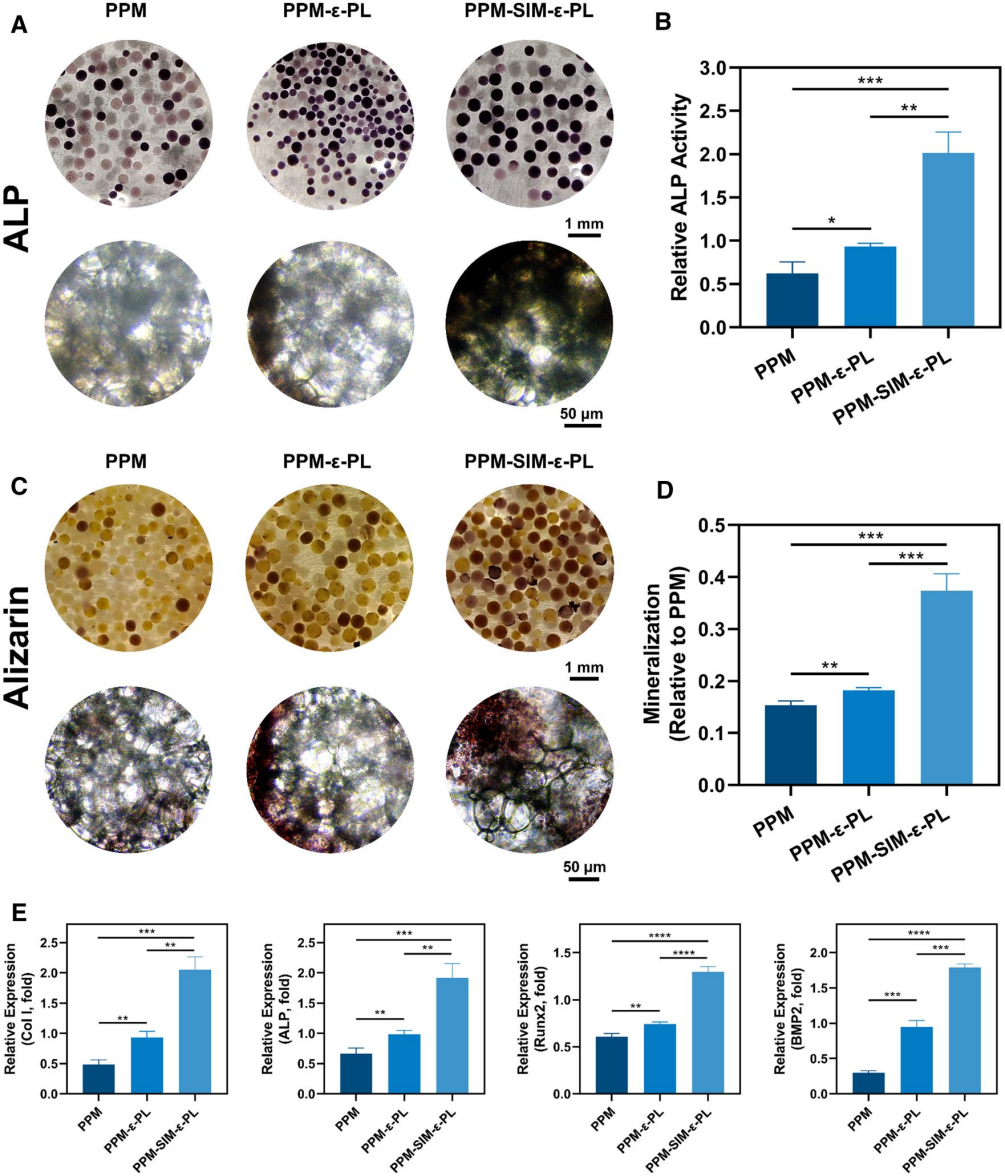
*In vitro* mineralization studies of cell-material composites. (**A**) ALP staining images of three sets of cell-material composites 14-day post-induction. (**B**) Quantitative assessment of ALP activity (*n* = 3). (**C**) Alizarin Red staining images of three sets of cell-material composites 21-day post-induction. (**D**) Quantitative evaluation of calcium nodule content at 21 days (*n* = 3). (**E**) mRNA expression of concerning osteogenic genes (Col I, ALP, Runx2 and BMP2) in different groups at 14 days.

Given the success of living materials construction, the regulation of MC3T3-E1 within living materials was further detected. At a 5-day culture of the osteoblast-microsphere complexes, the osteogenic medium was used for a substitutional culture, followed by the investigation of cell differentiation and mineralization. The ALP, a key indicator of early osteogenic differentiation and mineralization, was assessed at day 14, and the results revealed that the ALP expression within the PPM-SIM-ε-PL group increased significantly than that of the PPM and PPM-ε-PL group (Figure 7A and B). Moreover, regarding the influences of culture duration on osteogenic differentiation, we further extended culture time to 21 days to assess late osteogenic mineralization [46, 47]. Alizarin Red S staining at 14 and 21 days revealed a time-dependent increase in mineralization, and the PPM-SIM-ε-PL group exhibited noticeable calcium deposition levels at both times, with higher improvements by day 21 (Figure 7C and D; Figure S12). Additionally, Von Kossa staining performed on day 21 further reconfirmed the extensive mineralization in the PPM-SIM-ε-PL group (Figure S13).

To further elucidate the mechanism during the osteogenic differentiation, the expression of osteogenesis-related genes (Col I, ALP, Runx2 and BMP2) of the cell-microsphere complexes in groups was examined by quantitative PCR. After 14 days of osteogenic induction, compared with PPM and PPM-ε-PL groups, the PPM-SIM-ε-PL group exhibited significantly higher expression levels of all four genes, indicating a robust enhancement of osteogenic differentiation (Figure 7E). Moreover, the PPM-ε-PL group demonstrated significantly elevated expression levels relative to the PPM group, suggesting a positive effect of the ε-PL coating on osteogenic gene expression. These findings corroborated the potent promoting effect of SIM on osteogenic differentiation, further presenting synergistic osteogenic effects of ε-PL. The increased osteogenic markers demonstrated an improved MC3T3-E1 regulation via PPM-SIM-ε-PL, indicating the success of osteogenic differentiation at a molecular level.

**Figure 8.**
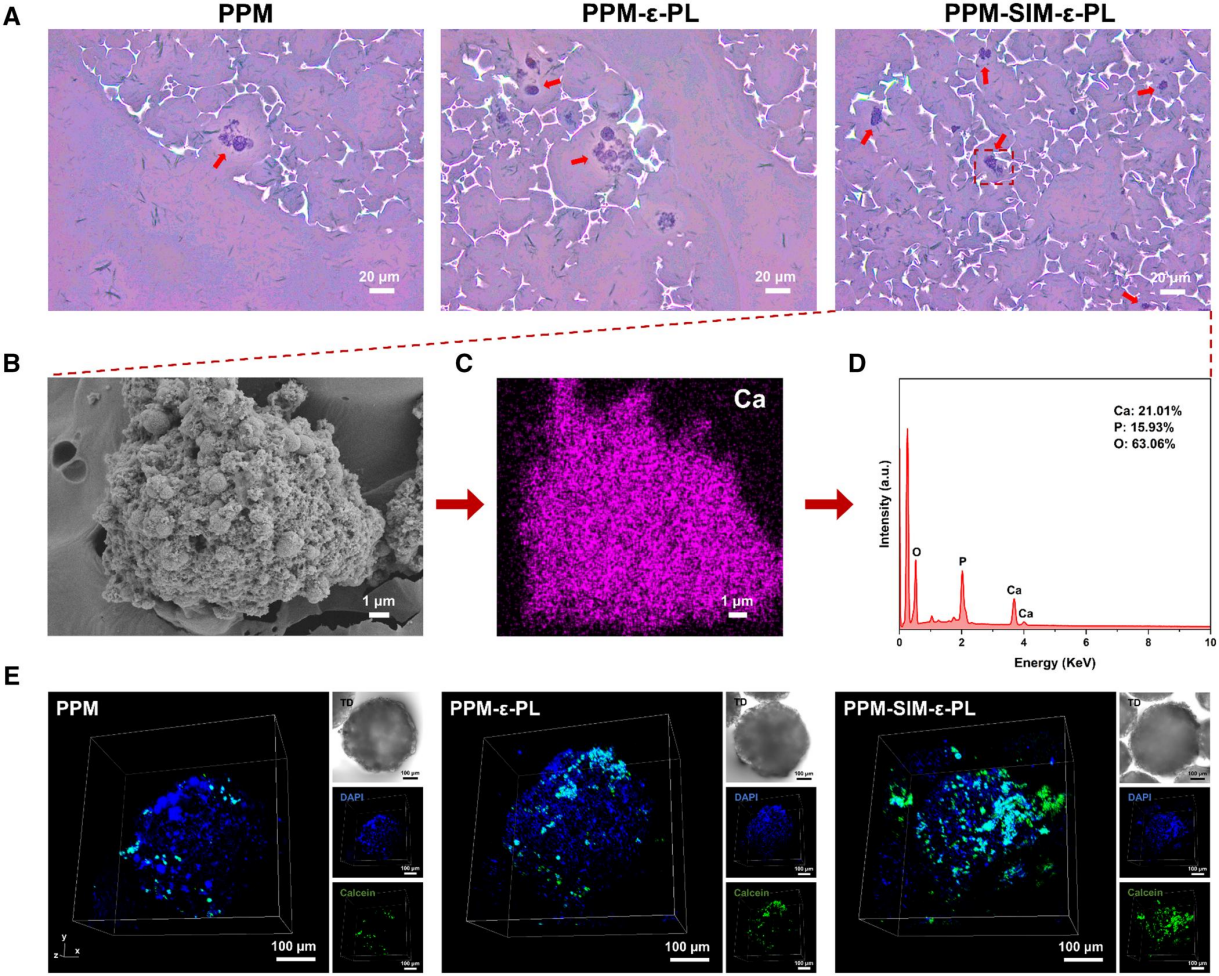
(**A**) H&E staining images of three sets of cell-material composite sections after 14 days of co-culture. (**B**) SEM imaging, (**C**) mapping analysis and (**D**) EDS elemental quantification of surface cells from the PPM-SIM-ε-PL group after 14 days of co-culture. (**E**) Calcium staining CLSM imaging of three cell-material composites after 14 days of co-culture.

After 14 days of co-culture, compared with PPM and PPM-ε-PL groups, the deep purple of H&E results within the PPM-SIM-ε-PL group indicated a significantly increased mineralization density (Figure 8A). Moreover, SEM and Ca element analysis demonstrated the typical cell matrix mineralization (Figure 8B–D), similar to the process *in vivo* [48], presenting a mimicry way of regulating osteocyte mineralization *ex vivo*. Furthermore, the presence of calcium phosphate deposition in the SIM-loaded composites was confirmed by the peaks spitting at 564 and 604 cm^−1^ in the infrared spectra [49] (Figure S14A and B) and material weight loss rate in the TGA (Figure S14C). Then, calcein, as a typical fluorescent dye binding to calcium mineral, was utilized to evaluate density mineralization [50], and the fluorescence intensity of calcium within PPM-SIM-ε-PL microspheres improved significantly than that of the other two groups (Figure 8E, Figure S15), which was consistent with the Alizarin Red Staining (Figure 7C). These achievements accelerated the PPM-SIM-ε-PL for MC3T3-E1 proliferation and functional regulation by releasing SIM, indicating the success of osteoblast-osteocyte shift regulation and mineralization at a microtissue level, presenting a promising boost osteoblast mineralization model by cell-microspheres integration, which would be of great potential for further bone regeneration.

### Subcutaneous ectopic osteogenesis of PPM

**Figure 9.**
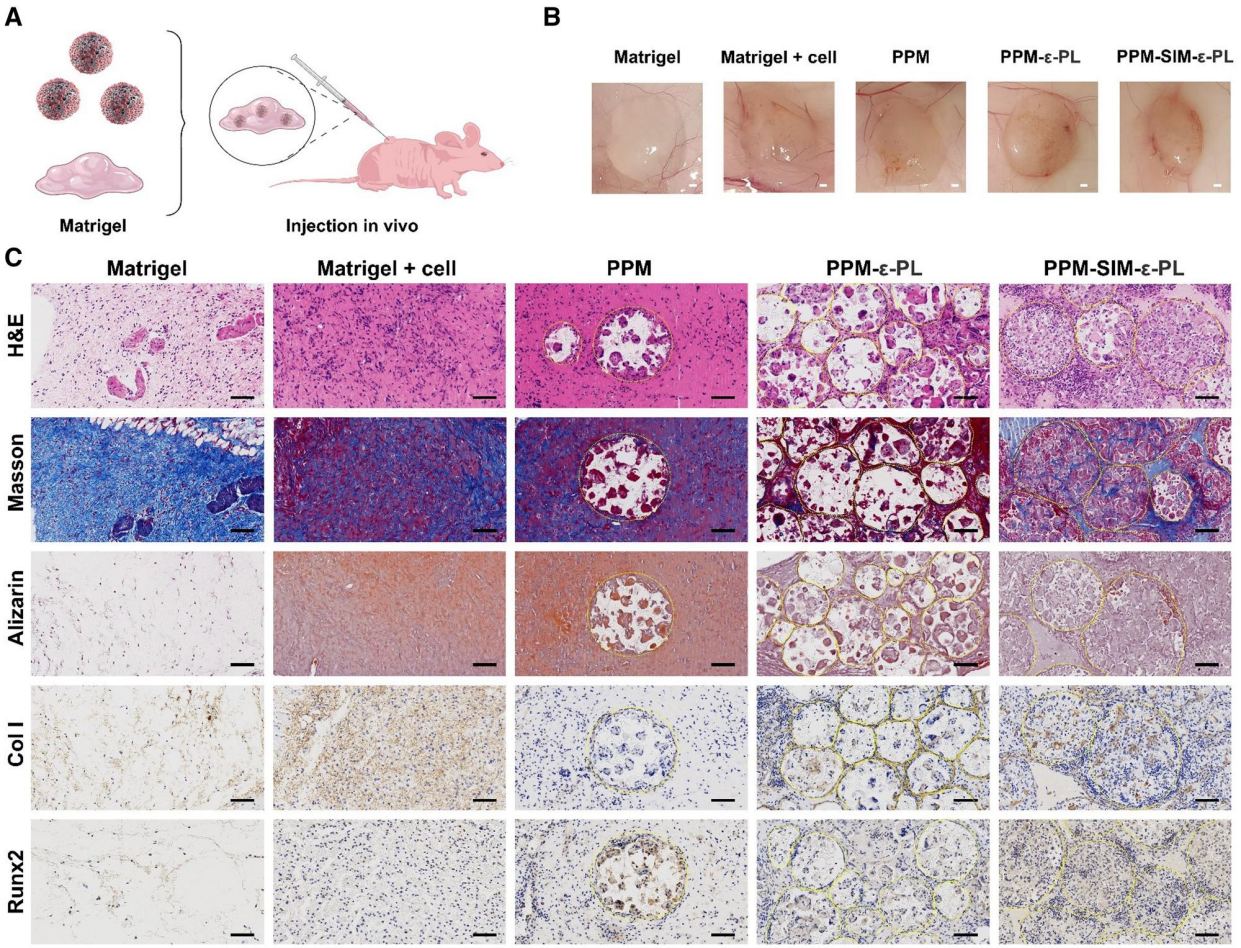
Subcutaneous ectopic osteogenesis of PPM. (**A**) Subcutaneous injection experiment schematic. Created with BioRender. (**B**) Subcutaneous tissue imaging 4-week post-transplantation. Scale bar = 1 mm. (**C**) Representative images of H&E staining, Masson’s trichrome staining, Alizarin Red S staining and immunohistochemical staining of Col I and Runx2 in different groups; the area of microsphere was outlined by a dotted line. Scale bar = 100 μm.

To verify that PPM had application potential in minimally invasive injection and bone repair, subcutaneous injection and assessment of osteogenic ability were performed (Figure 9A). Four-week post-injection, the implantation sites were examined optically (Figure 9B). All groups have no visible inflammatory responses, indicating the biocompatibility of microsphere complexes. Moreover, histological and immunohistochemical analyses of the implants further revealed cell differentiation and ossification (Figure 9C). H&E staining showed that the PPM-SIM-ε-PL group had the most abundant cell proliferation and tissue formation compared with the PPM and PPM-ε-PL groups. Masson’s trichrome staining demonstrated markedly enhanced collagen deposition in the PPM-SIM-ε-PL group, indicative of advanced extracellular matrix formation. Further, the calcium mineralization by Alizarin Red S staining revealed its great potential for osteogenesis of the PPM-SIM-ε-PL group, reconfirming its robust mineralization regulation. Immunohistochemical analysis of Col I and Runx2 further corroborated these findings. The PPM-SIM-ε-PL group exhibited more Col I and Runx2 expression, typically demonstrating a boosted mineralization process. The incorporation of SIM demonstrated a crucial role in promoting osteogenesis, while the ε-PL coating alone did not confer significant additional benefits over uncoated PLLA microspheres in this subcutaneous model. Collectively, these results indicated that the PPM-SIM-ε-PL microspheres possess superior osteogenic potential *in vivo*, significantly enhancing ossification compared with other groups.

## Conclusion

In conclusion, microfluidic technology was employed to develop porous PLLA microspheres, and the impact of diverse preparation parameters on the microspheres’ morphology was meticulously examined. Subsequently, 1% NH_4_HCO_3_ concentration with a homogenization rate of 5000 rpm and 2.5% PLLA with a 1:3 water-oil ratio was conducted on large-pore microspheres (>15 μm). The rationally designed porous PLLA microspheres were subsequently employed to culture MC3T3-E1 cells. This facilitated enhanced cell adhesion and proliferation and further regulated their biomineralization process *in vitro* and *in vivo*, forming the cell-material living composites. More importantly, osteoblast-based PPM-SIM-ε-PL living materials could improve osteocyte differentiation, accelerating the following osteogenesis and mineralization regulation *in vitro* and *in vivo*, boosting osteoblast mineralization by osteoblast-microspheres integration at the microtissue level, which presented an alternative *ex vivo* model involving osteocyte mineralization *in situ*, further indicating the success of cell-based living materials construction at microtissue levels.

## Supplementary Material

rbae125_Supplementary_Data

## Data Availability

The datasets supporting the conclusions of this article are included within the article and its additional files.
