# Supplementary material for: Cell-microsphere based living microhybrids for osteogenesis regulating to boosting biomineralization
Source: Regen Biomater. 2024 Oct 29;11:rbae125. doi: 10.1093/rb/rbae125 (PMC11578599; doi:10.1093/rb/rbae125)
Supplement: rbae125_Supplementary_Data [file rbae125_supplementary_data.docx]

**Supporting Information for**

**Cell-microsphere based Living Microhybrids for Osteogenesis Regulating to Boosting Biomineralization**

Zhaofan Hu^1,2#^, Yunyang Zhang^1,2#^, Jingjing Zhang^1,2^, Ran Zheng^1,2^, Yang Yang^1,2^, Fei Kong^1,2^, Haoran Li^1,2^, Xinyan Yang^3^, Shuhui Yang^1,2^, Xiangdong Kong^1,2^, Ruibo Zhao^1,2,4*^

^1^ Institute for Smart Biomedical Materials, School of Materials Science & Engineering, Zhejiang Sci-Tech University, Hangzhou 310000, PR China

^2^ Zhejiang-Mauritius Joint Research Center for Biomaterials and Tissue Engineering, Zhejiang Sci-Tech University, Hangzhou 310018, PR China

^3^ School of Laboratory Medicine and Bioengineering, Hangzhou Medical College, Hangzhou 311399, China

^4^ Zhejiang Sci-Tech University Shengzhou Innovation Research Institute, Shengzhou 312451, Zhejiang, China

**^___________________________________________________________________________________________________________________^**

**^#^** The authors contributed equally to this work.

**^*^**Corresponding author: Ruibo Zhao, [rzhao@zstu.edu.cn](mailto:rzhao@zstu.edu.cn)

**Table S1.** Primer sequences for qRT-PCR analysis.

| Target Gene Primer Sequence | | |
| --- | --- | --- |
| Col I | Forward | 5’-CCTTCTGGACCCGTTGGCAAAGAT-3’ |
|  | Reverse | 5’-GGCTACCCTGAGAACCACGAACA-3’ |
| ALP | Forward | 5′-ACCTGACTGACCCTTCGCTCT-3′ |
|  | Reverse | 5′-CAATCCTGCCTCCTTCCACCA-3′ |
| Runx2 | Forward | 5’-CACCATGCACCACCACCTCGAAT-3’ |
|  | Reverse | 5’-GCTTCCGTCAGCGTCAACACCAT-3’ |
| BMP2 | Forward | 5’-GGACACCAGGTTAGT GAATCAGA-3’ |
|  | Reverse | 5’-CTTCCACCACAAACCCATGGTT-3’ |
| GAPDH | Forward | 5’-GAAGGTCGGTGTGAACGGATTTG-3’ |
|  | Reverse | 5’-CATGTAGACCATGTAGTTGAGGTCA-3’ |

**Table S2.** Different formulation parameters for preparing PPM.

| Group | NH_4_HCO_3_ Concentration (%) | Homogenization Rate (rpm) | W/O proportion | PLGA Concentration (%) |
| --- | --- | --- | --- | --- |
| 1% NH_4_HCO_3_ | 1 | 10000 | 1:3 | 2.5 |
| 5% NH_4_HCO_3_ | 5 | 10000 | 1:3 | 2.5 |
| 10% NH_4_HCO_3_ | 10 | 10000 | 1:3 | 2.5 |
| 5000 rpm | 1 | 5000 | 1:3 | 2.5 |
| 10000 rpm | 1 | 10000 | 1:3 | 2.5 |
| 20000 rpm | 1 | 20000 | 1:3 | 2.5 |
| 1% PLLA | 1 | 5000 | 1:3 | 1 |
| 2.5% PLLA | 1 | 5000 | 1:3 | 2.5 |
| 4% PLLA | 1 | 5000 | 1:3 | 4 |
| 1:2 | 1 | 5000 | 1:2 | 2.5 |
| 1:3 | 1 | 5000 | 1:3 | 2.5 |
| 1:4 | 1 | 5000 | 1:4 | 2.5 |

**
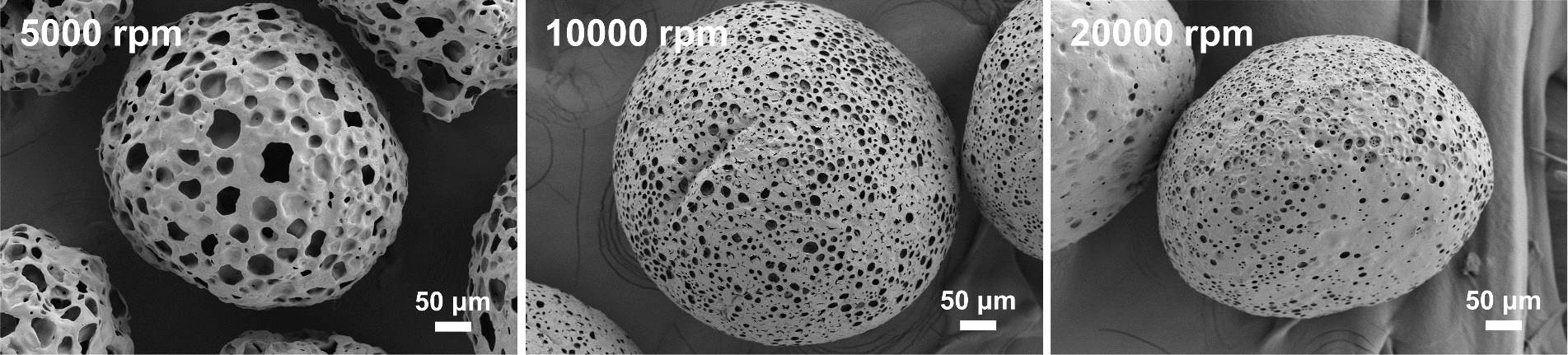
**

**Figure S1****.** SEM images of PPM produced with different homogenization rates.


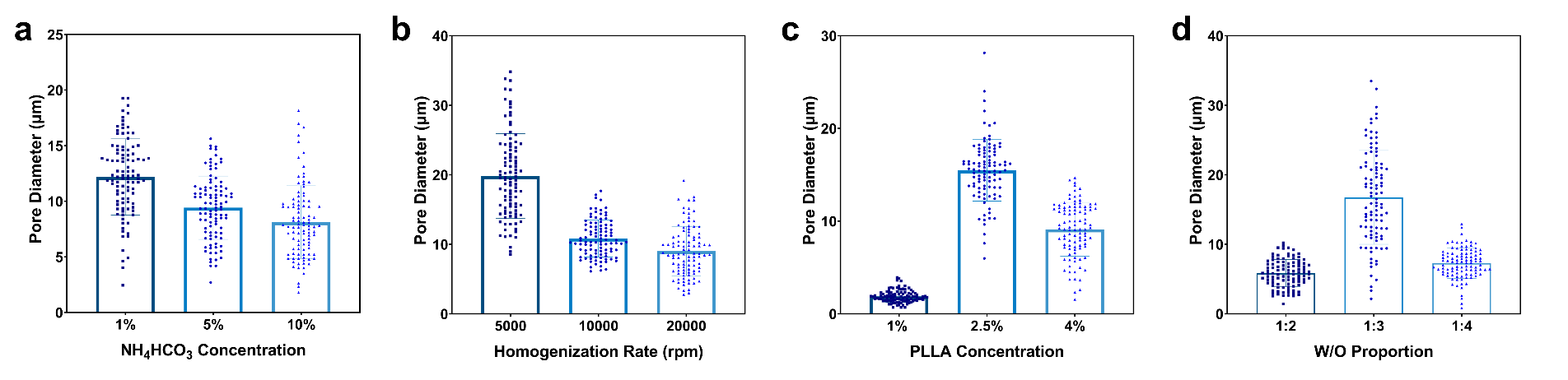


**Figure** **S2.** Pore size distribution of PPM in different groups. Measurement of pore size was based on the SEM images (n=100).


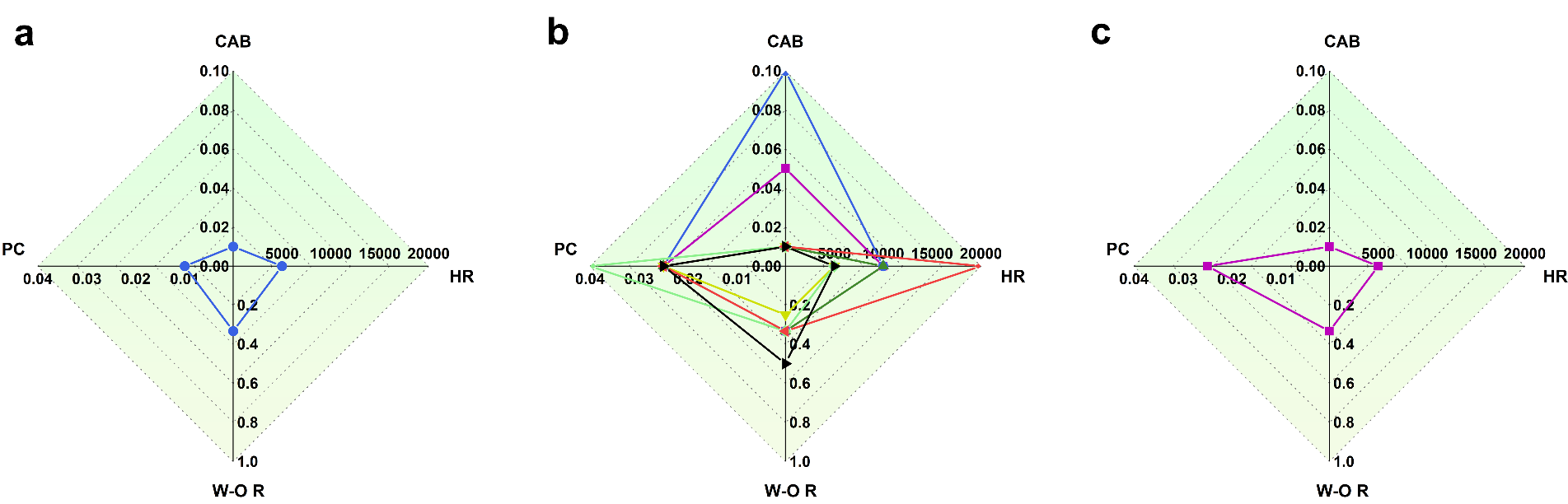


**Figure S3.** (a-c) Radar map of the preparation parameters of porous microspheres with pore sizes (< 5μm; 5-15μm; > 15μm). (CAB: Concentration of ammonium bicarbonate (w/v), HR: Homogenization Rate (rpm), W-O R: Water-oil Ratio, PC: PLLA Concentration (w/v)).

*
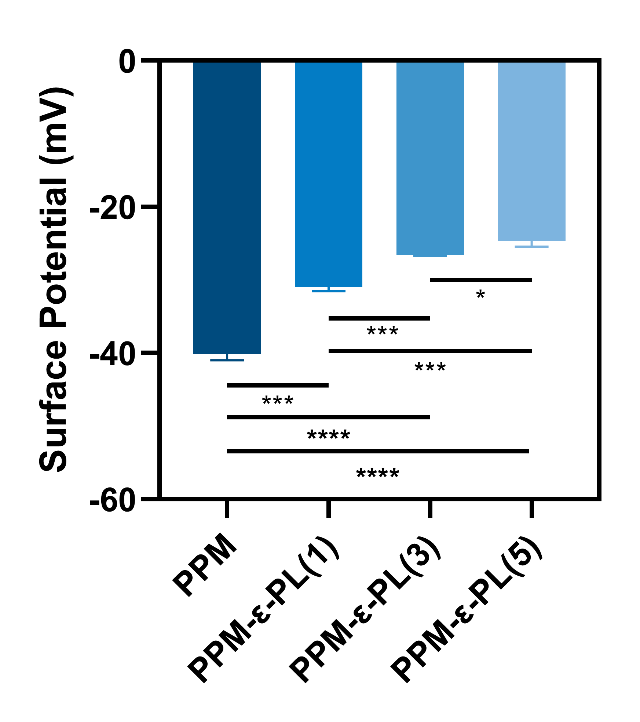
*

**Figure S4.** Surface potential measurements of PPM modified with different concentrations of ε-PL.


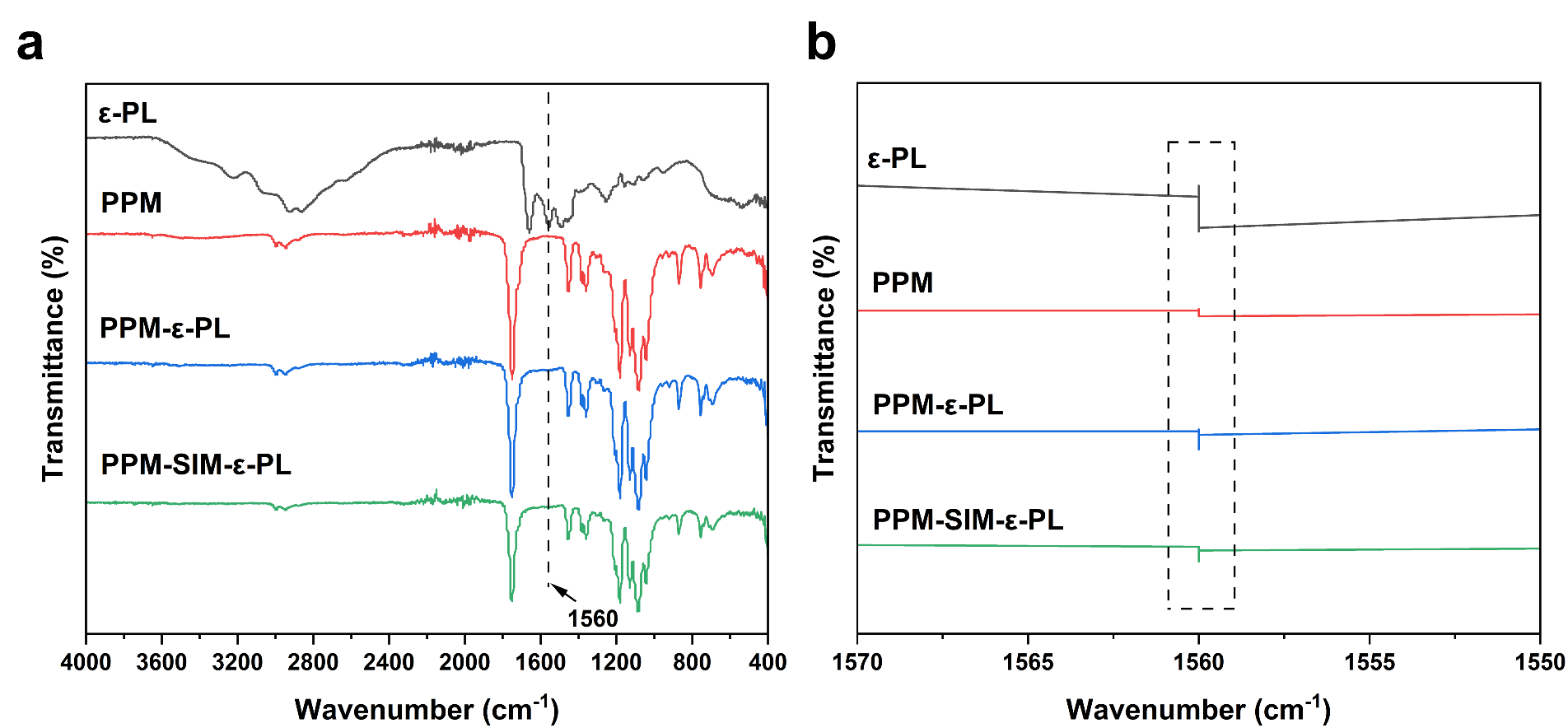


**Figure S5.** (a) FTIR spectra of ε-PL, PLLA, PPM-ε-PL, and PPM-SIM-ε-PL. (b) Detailed analysis of the spectra focusing on the wavenumber at 1560 cm^-1^.


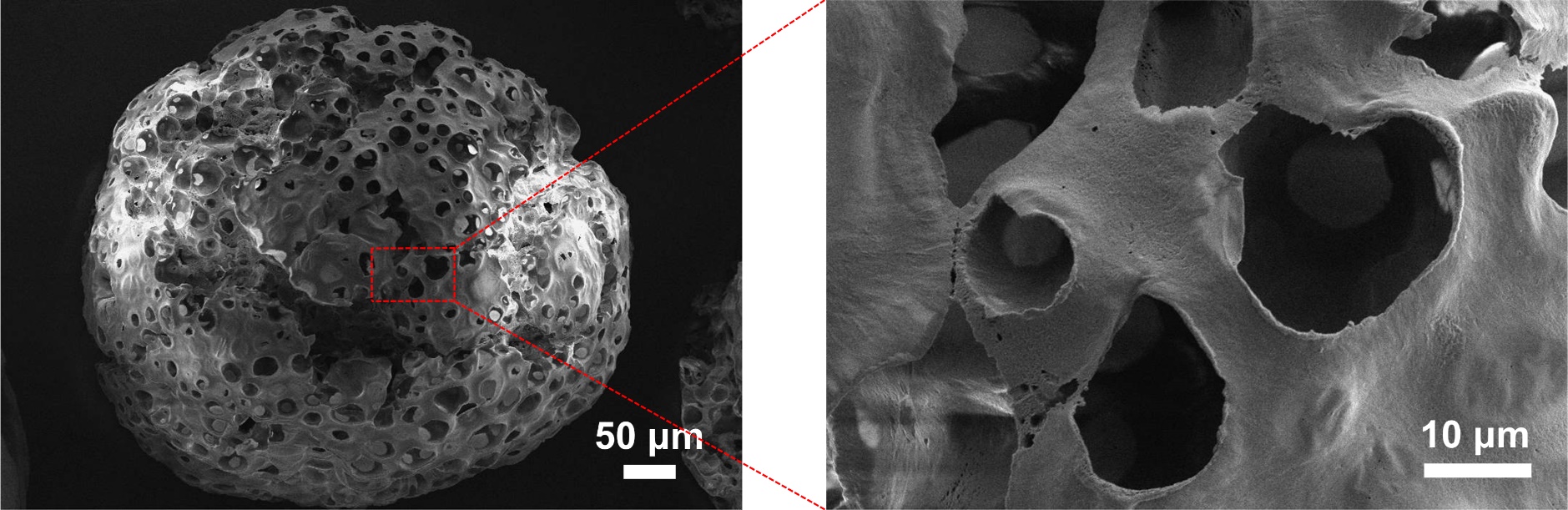


**Figure S6.** SEM images of PPM-SIM-ε-PL (with high drug concentration).


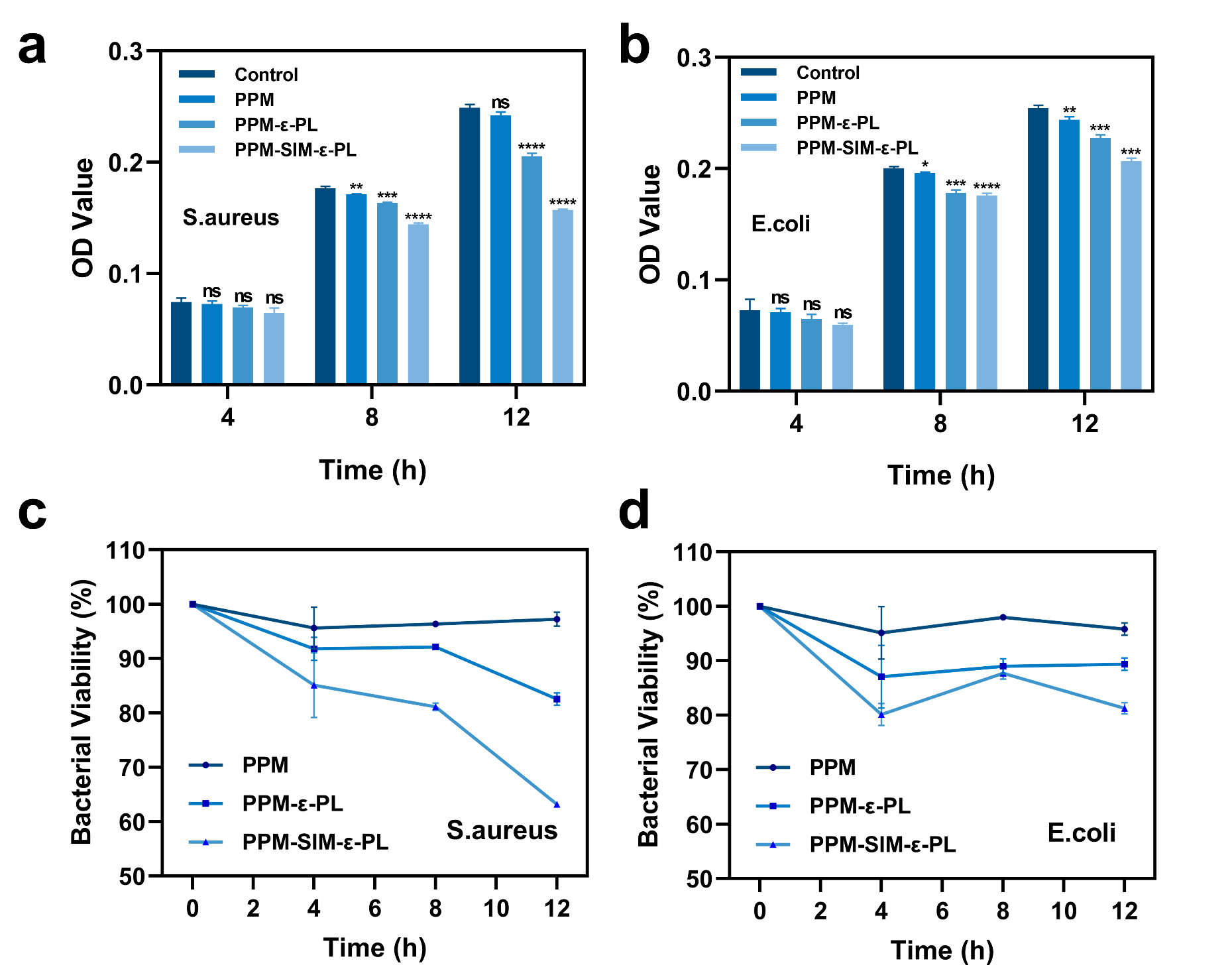


**Figure S7.** In vitro antibacterial activity of microspheres against S. aureus and E. coli. Optical density (OD) values of S. aureus (a) and E. coli (b) suspensions co-cultured with different microspheres for 4, 8, and 12 h. Bacterial viability of S. aureus (c) and E. coli (d) after co-culture with microspheres for 4, 8, and 12 h.


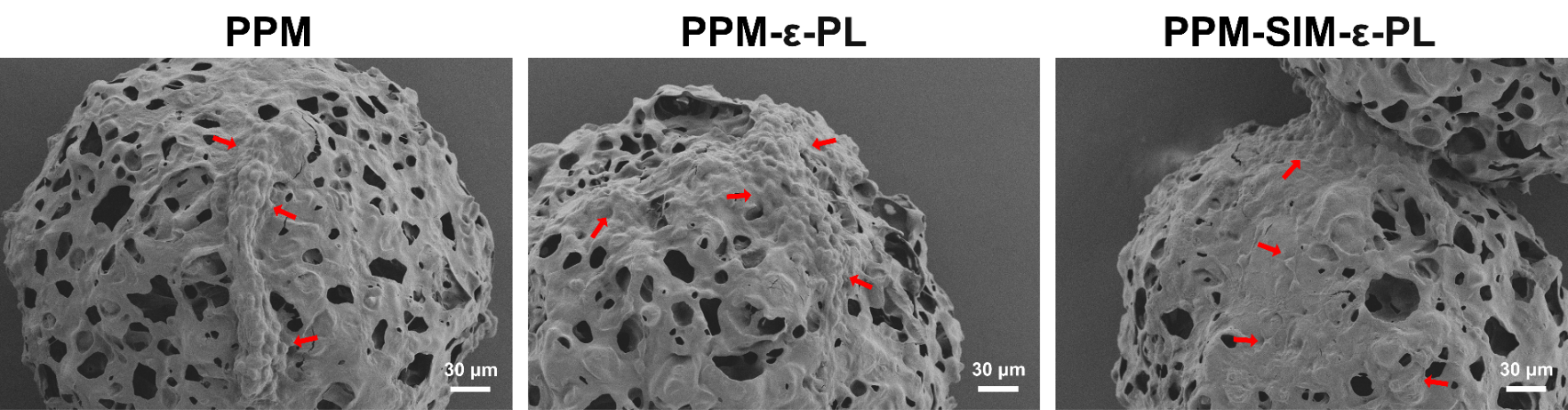


**Figure S8.** Cell growth on microspheres after 3 days of co-culture.


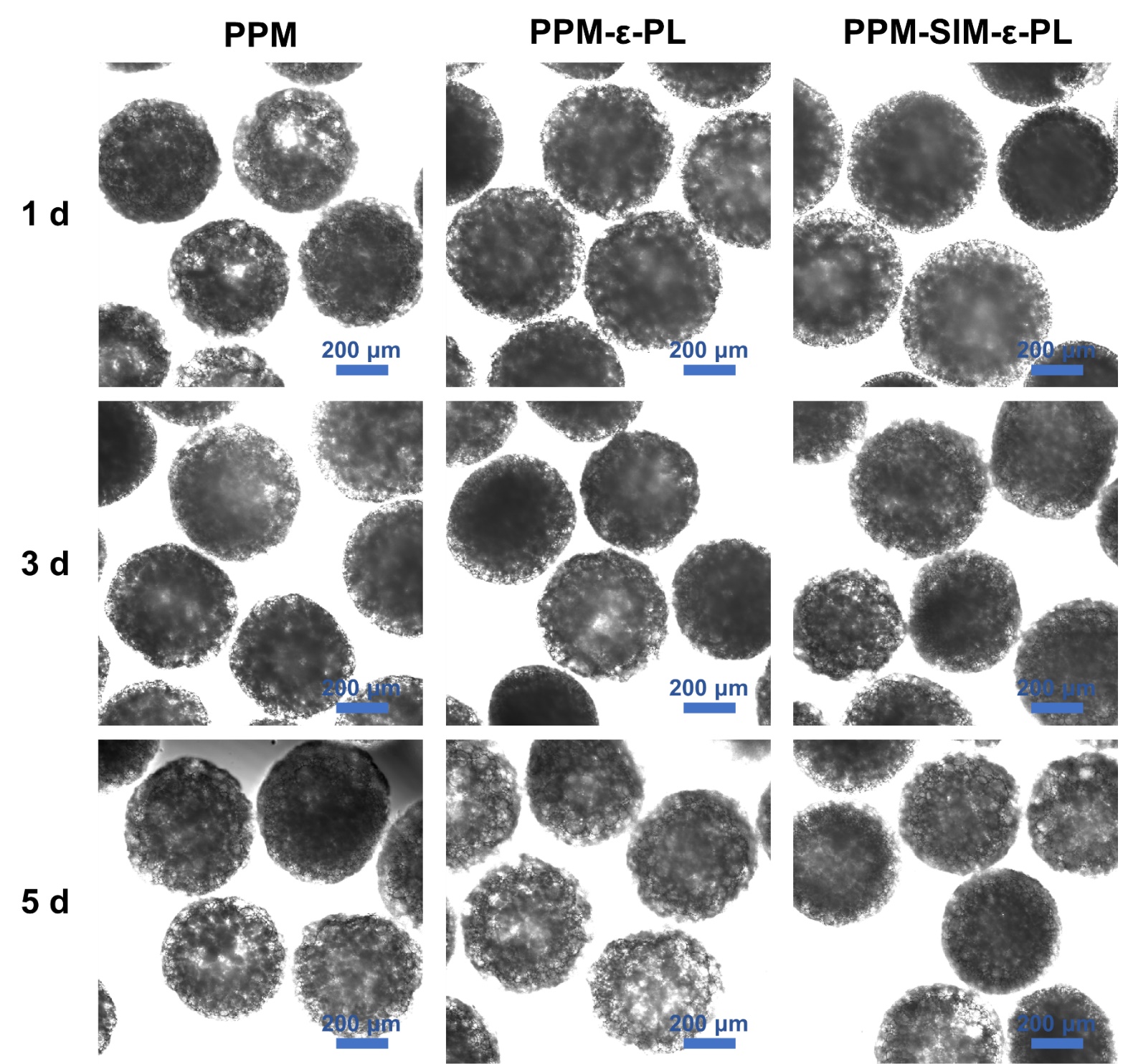


**Figure S9.** Bright-field images of MC3T3-E1 cells cultured on different groups at 1, 3, and 5 days.

**
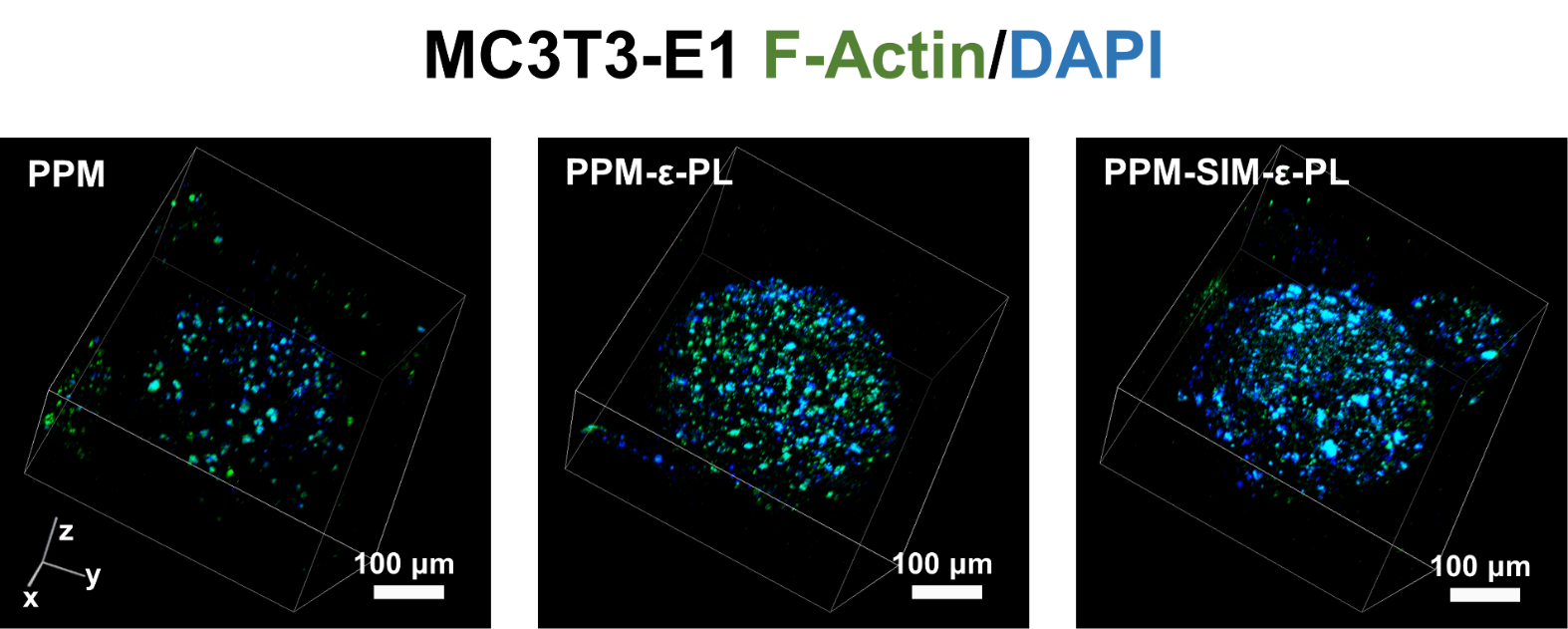
**

**Figure S10.** Fluorescence images of MC3T3-E1 cells adhering to different microsphere sets after 5 days of co-culture. Cells were stained with F-actin (green) and DAPI (blue).


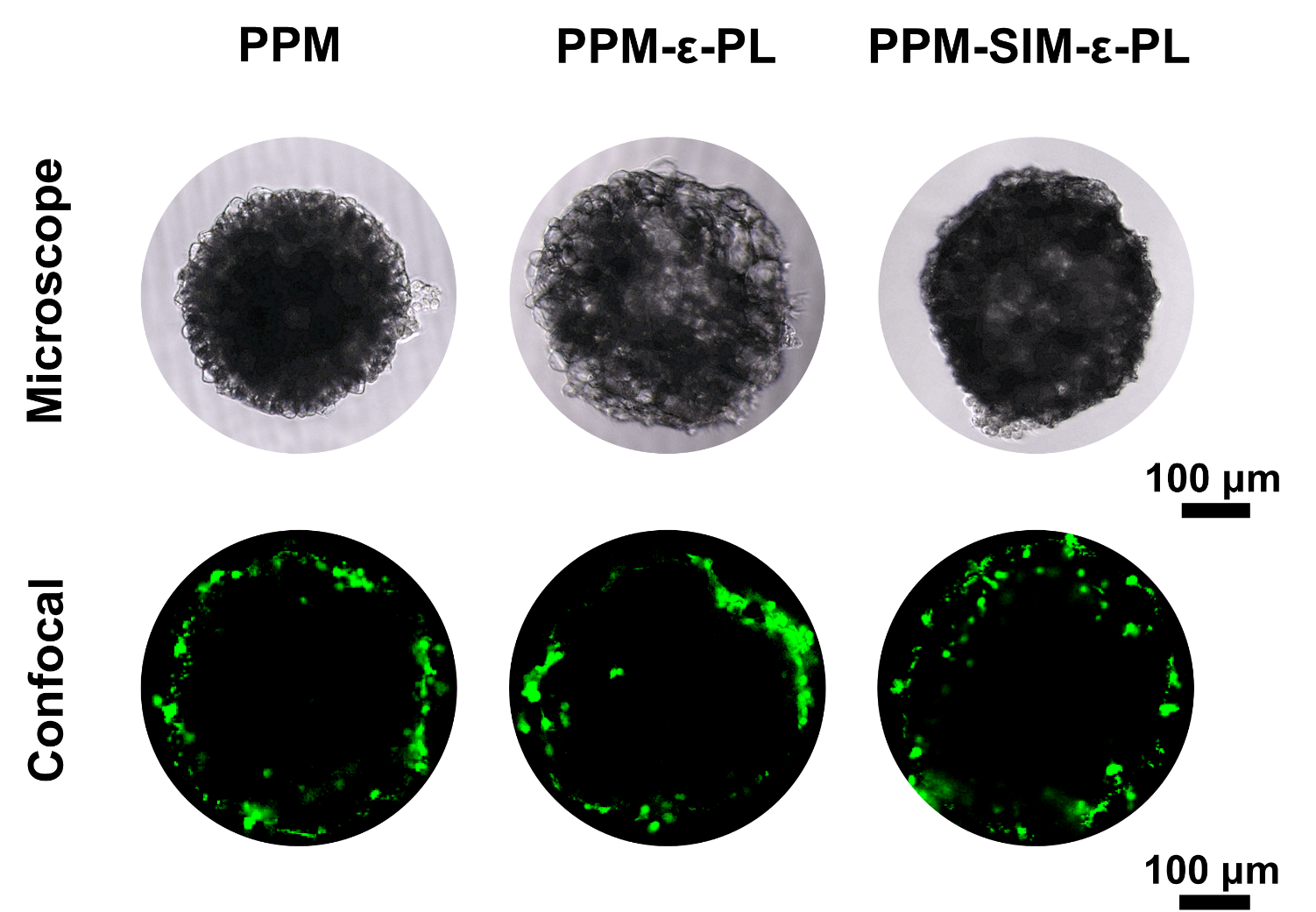


**Figure S11.** Microscopy and live/dead staining confocal imaging of cell-microsphere complexes in 24-well plates.


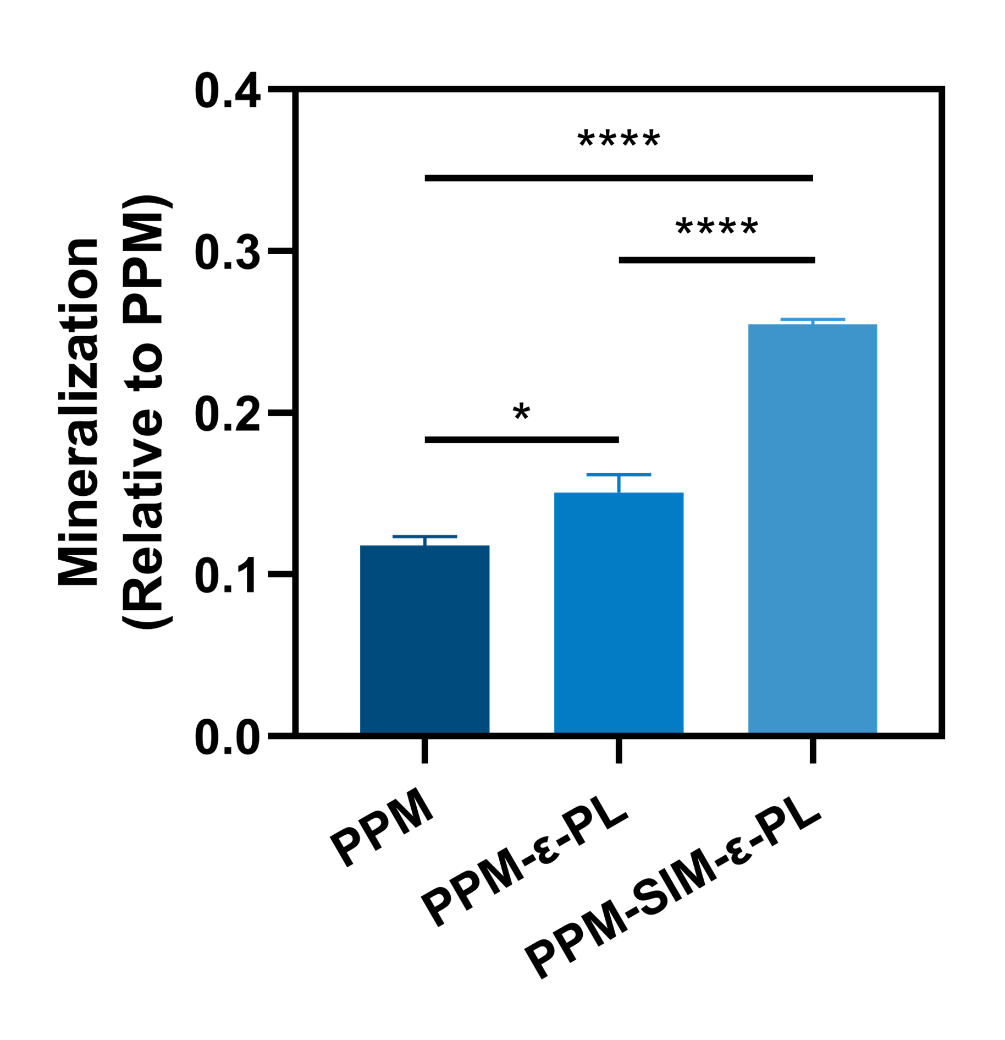


**Figure S12.** Quantitative evaluation of calcium nodule content at 14 days.

**
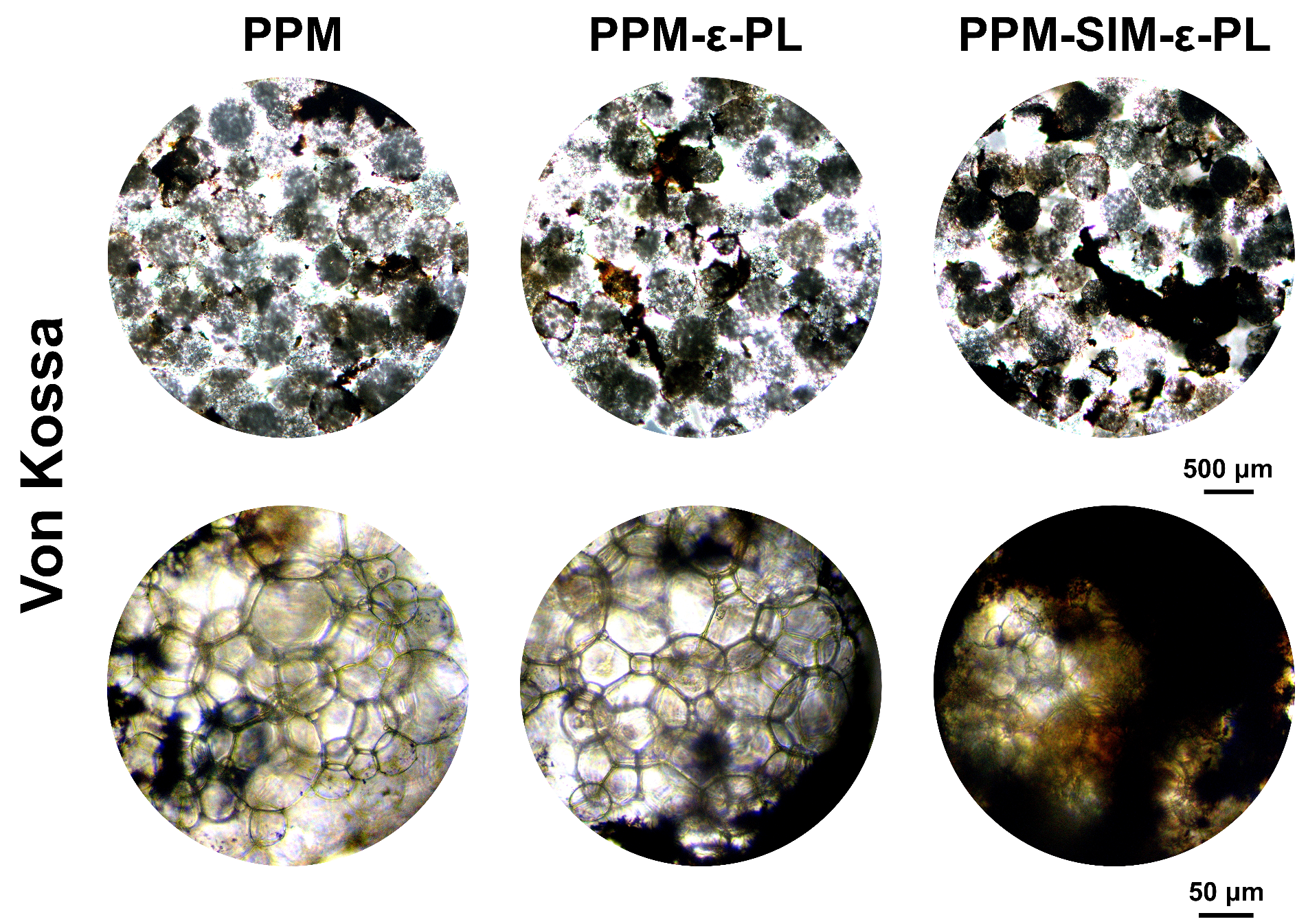
**

**Figure S13.** Von Kossa staining images of three sets of cell-material composites 21 days post-induction.


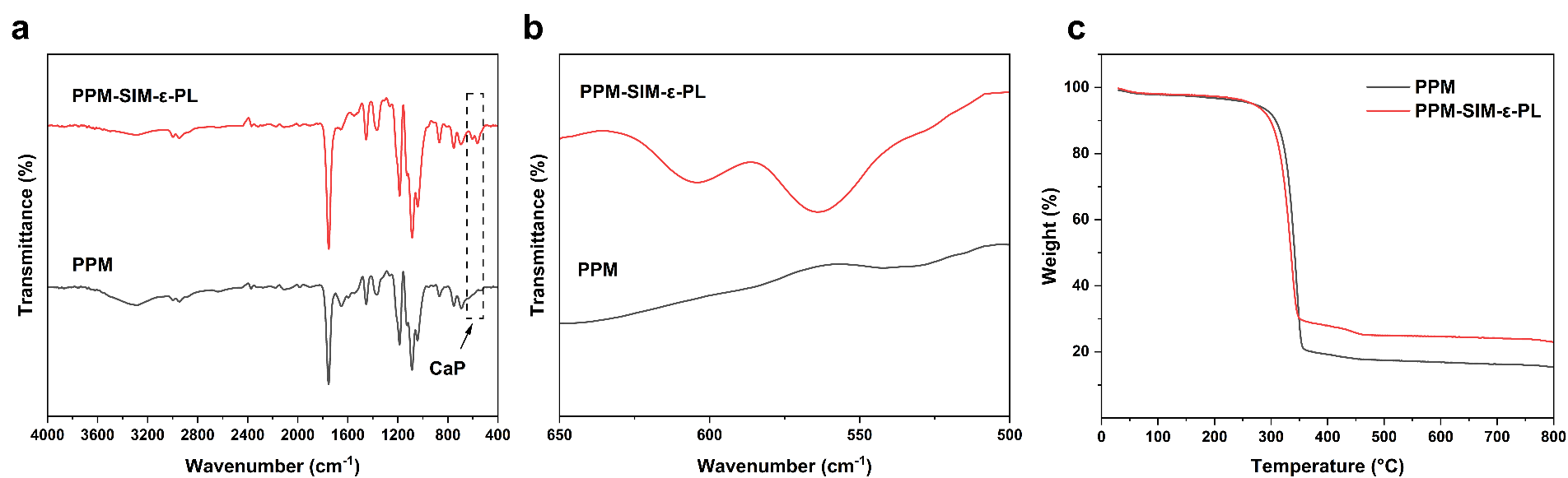


**Figure S14.** Fourier transform infrared spectroscopy (FTIR) profiles (a-b) and Thermalgravimetric analysis (c) of the composites after 14 days of co-culture with MC3T3-E1 cells.


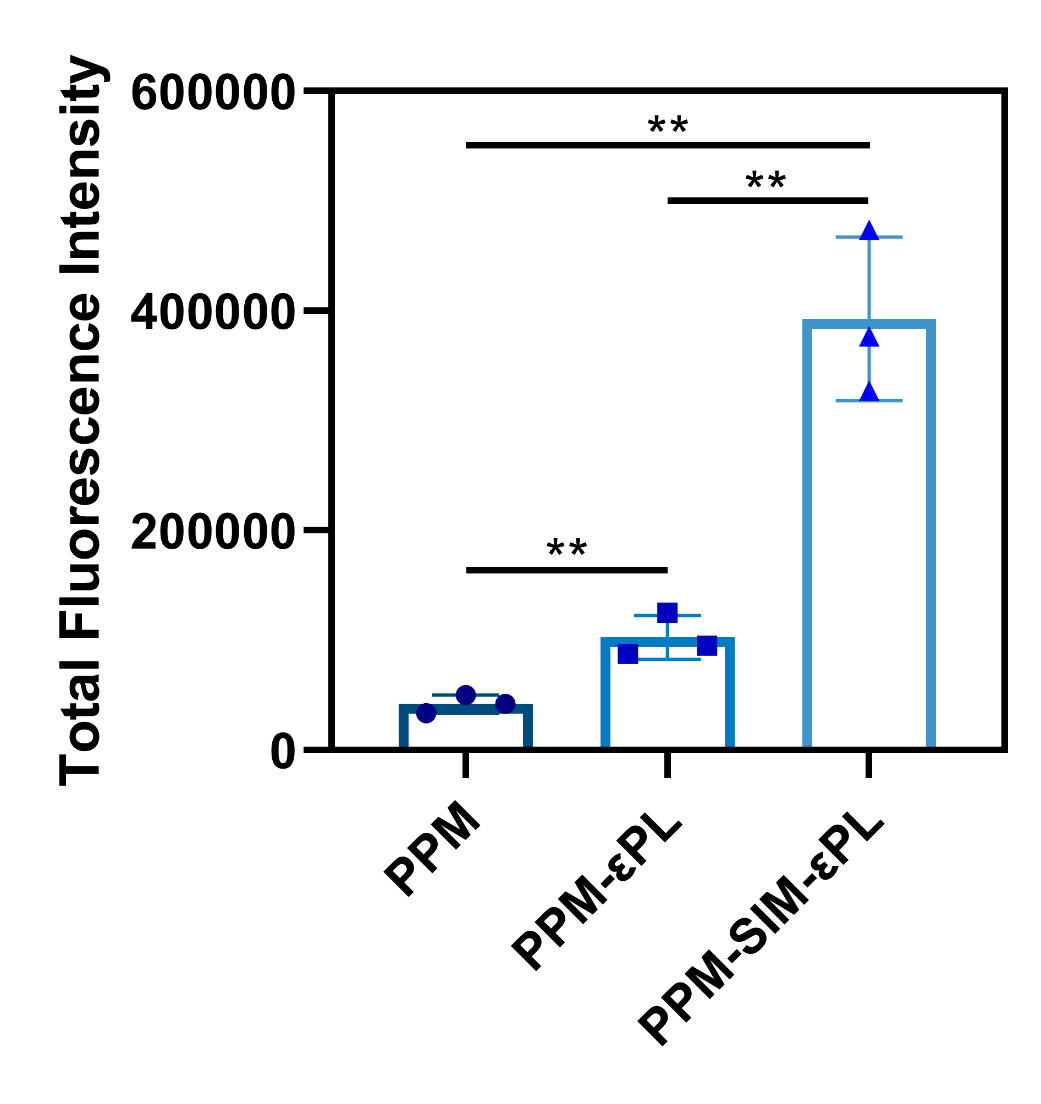


**Figure S15.** Quantitative results of total fluorescence intensity of calcein staining of composites (n = 3).
